# Barriers and Facilitators to Patient Acceptance of Artificial Intelligence in Health Care: Systematic Review

**DOI:** 10.2196/80581

**Published:** 2026-05-08

**Authors:** Huiqin Shi, Jingying Huang, Jin Yang, Mengbo Han

**Affiliations:** 1 Department of Nursing, Intensive Care Unit, Sir Run Run Shaw Hospital, Zhejiang University School of Medicine, No. 3 East Qingchun Road, Hangzhou, Zhejiang, 310016, China, +86 177 5717 3794; 2 Department of Nursing, Sir Run Run Shaw Hospital, Hangzhou, China

**Keywords:** artificial intelligence, patients, barriers, facilitators, interventions

## Abstract

**Background:**

Artificial intelligence (AI) in the domain of health care is increasing in prominence. Acceptance is an indispensable prerequisite for the widespread implementation of AI.

**Objective:**

This study aimed to explore barriers and facilitators influencing patients’ acceptance of AI.

**Methods:**

We conducted a systematic review following PRISMA (Preferred Reporting Items for Systematic Reviews and Meta-Analyses) guidelines. Nine databases, including PubMed, Web of Science, and Embase, were comprehensively searched from inception to December 23, 2025. We included qualitative, quantitative, and mixed methods studies investigating adult patients’ attitudes toward medical AI. Two researchers independently screened records, extracted data, and appraised methodological quality using the Mixed Methods Appraisal Tool. Following the Joanna Briggs Institute convergent integrated approach, data synthesis was guided by integrating the Unified Theory of Acceptance and Use of Technology 2 (UTAUT2) and the Theoretical Domains Framework (TDF). Factors were mapped to behavior change techniques (BCTs) and evaluated for practical feasibility using the Affordability, Practicability, Effectiveness and cost-effectiveness, Acceptability, Side-effects/safety, and Equity criteria.

**Results:**

A total of 61 studies met the inclusion criteria out of 7452 search results. Study designs included qualitative (n=20), quantitative (n=35), and mixed methods (n=6). Performance and effort expectancies were the primary determinants of acceptance. Major barriers comprised perceived operational complexity, lack of algorithmic trust, reduced interpersonal interaction, privacy vulnerabilities, and high costs. Facilitators included transparent data governance, interpretability of AI decisions, improved clinician-patient communication, and human-centered design. Education level and disease severity emerged as key moderating variables. Through UTAUT2-TDF mapping, we identified 25 distinct BCTs (6 high, 14 medium, and 5 low feasibility) and formulated 40 actionable intervention strategies.

**Conclusions:**

This study innovatively integrates the UTAUT2 and TDF frameworks to evaluate patient acceptance of medical AI. Unlike existing reviews that predominantly evaluate isolated psychosocial factors or purely technical attributes, this transtheoretical approach differentiates itself by merging technology adoption mechanisms directly with behavioral drivers. Consequently, it contributes to the field by systematically identifying multilevel factors influencing acceptance, including performance expectancy, effort expectancy, and ethical security, and translating these into 40 actionable BCTs. In real-world clinical practice, these findings provide a feasible, prioritized blueprint for clinicians and administrators to design patient-centered interventions, enhancing the clinical integration and long-term effectiveness of medical AI.

## Introduction

Artificial intelligence (AI) is a transformative technology that integrates machine learning and deep learning, demonstrating vast potential across various medical and scientific fields [1]. AI in medicine relies on technologies such as machine learning and natural language processing to transform medical imaging analysis, clinical decision support, and personalized treatment planning, thereby enhancing diagnostic accuracy, efficiency, and patient outcomes [2,3]. By enhancing diagnostic efficiency and accuracy through intelligent medical image analysis, early disease risk prediction, and personalized treatment planning, AI has significantly improved patient outcomes and health care experiences [1]. AI has been integrated into the health care sector to offer effective, clinically relevant solutions for patients and medical professionals. An expanding body of literature has demonstrated that AI-based interventions can equal or even surpass physician expertise [4].

Nevertheless, its adoption on a broader scale remains limited. Ethical concerns, standardization gaps, and ambiguous legal responsibilities are among the challenges hindering the widespread implementation of AI in health care today. According to Rogers’ Diffusion of Innovations theory, user acceptance is pivotal in determining the adoption rate of new technologies [5]. In technological contexts, acceptance is characterized as the readiness, purpose, and intrinsic motivation to use a technology, stemming from positive attitudes toward the system [6]. The acceptance of AI systems follows a similar dynamic to other new tools, but the unpredictable management of complex scenarios and expectations for human-like interactions often amplify resistance. Accepting change is a complex process, as humans are often averse to altering the familiar status quo in favor of comfort. Yet, for enhancing patient outcomes and experiences throughout the health care journey in the long term, acceptance remains a crucial factor in adopting and integrating newly introduced innovations such as AI into daily practice [7]. Understanding patient acceptance is crucial for improving AI adoption, informing health care policy, and building a more robust and equitable health care system [8].

With the advancement of medical AI, its implementation in clinical practice has evoked complex attitudes and feelings among patients [9,10]. Therefore, many scholars have investigated the willingness to adopt such technology among different patient groups, aiming to identify relevant barriers and facilitating factors. Alsanosi et al [11] used questionnaire surveys to explore the awareness, attitudes, and behaviors toward AI among specific patient populations, finding that education level, age, and self-efficacy positively influenced their willingness to use it. Gundlack et al [12] conducted in-depth interviews through a semistructured approach with a subset of patients, revealing that patients were most concerned with whether AI could reduce medical costs, shorten waiting times, and accelerate the diagnostic and treatment process. Ensuring transparency in the development, data processing, and intended use of AI systems was also considered crucial [13]. Research by Longoni and Morewedge [10] highlights that, despite clear evidence of AI’s cost-effectiveness, accuracy, and operational efficiency, many patients remain hesitant about AI systems that operate autonomously or make critical medical decisions without human involvement.

Despite the valuable insights offered by previous studies, there remains a lack of systematic integration and supplementation grounded in a unified theoretical framework. This gap hinders the effective translation of research findings into practical clinical interventions. Given the complexity and multidimensionality of patient perceptions, applying a well-established theoretical model to synthesize existing evidence on the factors influencing AI adoption can not only elucidate the underlying mechanisms but also enhance the generalizability and applicability of research outcomes across diverse clinical contexts. Various theoretical models have been developed to measure the acceptance of technological innovations, such as the technology acceptance model and the Unified Theory of Acceptance and Use of Technology 2 (UTAUT2) [14]. These approaches have been widely adopted in prior research, including in the health care field, such as exploring patient adoption of mobile health apps [15], telemedicine technologies [16], and wearable devices [17], demonstrating strong predictive power in explaining patients’ behavioral intentions and actual usage behaviors. UTAUT2 integrates 8 technology acceptance-related theories, including the technology acceptance model, by synthesizing key factors influencing behavioral intention and usage behavior: performance expectancy, effort expectancy, social influence, and facilitating conditions [15,18]. It also incorporates moderating variables such as gender, age, voluntariness, and experience.

Therefore, this study uses the UTAUT2 framework to examine the direct determinants of patients’ acceptance of medical AI technologies from a technology adoption perspective. However, while this model effectively identifies influencing factors with an emphasis on technological attributes and user psychology, it offers limited explanatory power regarding specific behavior change mechanisms [19]. Furthermore, its theoretical dimensions remain relatively abstract, posing challenges for deriving targeted intervention strategies. To address these limitations, we integrate the Theoretical Domains Framework (TDF), a well-established framework in behavioral science, to supplement and refine the dimensions of UTAUT2 [20]. By leveraging the mapping relationships between theoretical domains and behavior change techniques (BCTs) [21], this integrative approach bridges technology acceptance theory and behavioral science, enabling the identification of tailored BCTs to guide clinical practice and enhance patient acceptance of AI. Building on this dual-theoretical foundation, the primary aim of this systematic review is to synthesize evidence through the combined lenses of technology acceptance and behavioral science to comprehensively identify the barriers and facilitators to patient acceptance of AI in hospital settings.

## Methods

### Overview

This review was reported following the PRISMA (Preferred Reporting Items for Systematic Reviews and Meta-Analyses) 2020 statement [22]. The protocol was registered with PROSPERO in October 2024 (registration no CRD42024598884). A theoretically grounded mixed methods systematic review provides a structured and integrative approach to identifying the barriers and facilitators that influence patient acceptance of AI.

### Search Strategy

The literature search was conducted following the PRISMA-S (Preferred Reporting Items for Systematic Reviews and Meta-Analyses extension for Searching) reporting guidelines [23]. Two researchers (HS and JY) and an evidence-based expert (JH) developed the search strategy using the Population, Interest, and Context framework [24]. Eligibility criteria focused on patients, AI artifacts, and attitudes and perceptions. Barriers and facilitators were defined as factors preventing or motivating AI use. The search strategy for this study was built around the core terms “artificial intelligence,” “patient,” and “attitude,” combining the use of medical subject headings and keywords. A comprehensive search was conducted across PubMed, Web of Science, Embase, Cochrane Library, CINAHL, CNKI, Wan Fang, VIP, and SinoMed (inception to December 23, 2025). Detailed search strategies are provided in Table S1 in Multimedia Appendix 1. Our comprehensive search strategy was executed across multiple sources to ensure literature saturation. This included searching major study registries, systematic online database searches, manual browsing of key journals and organizational websites, and citation searching (both backward and forward) of all included studies and relevant reviews. No restrictions were applied based on publication status or language during the initial search phase.

### Inclusion and Exclusion Criteria

We included qualitative, quantitative, and mixed methods studies that addressed (1) patients’ perceptions of using AI in health care (diagnosis and treatment) and (2) barriers and facilitators to patients’ willingness to use AI. Studies limited to health care professionals, managers, technology developers, and medical and nursing students, as well as studies that only tested the effectiveness of AI, were excluded. Table 1 shows the inclusion and exclusion criteria for this study.

**Table 1.** Inclusion and exclusion criteria for the mixed methods systematic review on patient acceptance of medical artificial intelligence.

	Population	Phenomena of interest	Context	Study type	Limits
Inclusion criteria	Patients (regardless of disease type) Patients aged ≥18 years	Patient perceptions of the use of AI^a^ (diagnostic and therapeutic). Barriers and facilitators to patients’ willingness to use AI	All medical fields	Qualitative studies Quantitative studies Mixed methods studies	Literature in English or Chinese
Exclusion criteria	Health care professionals, administrators, or technology developers only Students of medicine and nursing	Studies investigating the effectiveness of AI technology in health care only	Nonmedical scenarios such as sports and fitness	Gray literature Conference abstracts Unpublished studies and dissertations	—^b^

a AI: artificial intelligence.

b Not available.

### Literature Screening and Data Extraction

Two researchers (HS and MH) independently screened papers after removing duplicates in EndNote (version 21; Clarivate), resolving disagreements through discussion or third-party consultation (JH). Data on barriers, facilitators, and interventions were extracted using a data extraction table [24]. Qualitative studies provided themes and participant quotes, while quantitative studies included outcome measures and narrative summaries. Mixed methods studies were analyzed separately. Extracted details included author, year, design, participant characteristics, findings, and interventions, with open-ended survey responses treated as qualitative data, ensuring comprehensive extraction and synthesis. When necessary, corresponding authors were contacted to obtain missing or supplementary data.

### Quality Assessment

The Mixed Methods Appraisal Tool (MMAT) [25] was used to assess the methodological quality of the included studies, covering qualitative, quantitative (randomized/nonrandomized), and mixed methods designs. Two researchers (HS and MH) independently conducted assessments, resolving disagreements through consensus or consultation with a third expert (JH).

### Synthesis Methods

#### Overview

This study aims to systematically identify the barriers to and facilitators of patient adoption of AI in health care settings. Given that the research question is well-suited to be addressed through complementary qualitative and quantitative evidence, we adopted the “convergent integrated approach” from the Joanna Briggs Institute methodology for data synthesis [26]. This method involves transforming quantitative data into a qualitative format and integrating it with original qualitative findings, which are subsequently categorized and integrated based on similarity in data meaning. This procedure was initially performed by one researcher and then discussed and cross-checked with a senior researcher experienced in evidence-based practice.

We used narrative synthesis rather than meta-analysis due to substantial heterogeneity in the included studies regarding design, measurement, and AI application contexts [27]. This heterogeneity precluded a statistically meaningful quantitative pooling of effect sizes. Consequently, certain items on the Synthesis Without Meta-analysis checklist pertaining strictly to meta-analytical data transformations were not applicable to this study. Our primary objective was to explore the complex mechanisms influencing acceptance, an explanatory aim better served by narrative synthesis that integrates qualitative and quantitative evidence, rather than by quantifying isolated effect sizes.

#### Integrated Analytical Framework

During the thematic synthesis stage, the identified obstacles and facilitators were summarized by theme and mapped into the various domains of the UTAUT2 [28], which is a reliable framework for understanding technology acceptance [29-32]. To deepen the behavioral analysis, these factors based on UTAUT2 were further analyzed through the TDF [33]. The TDF is based on behavioral science and provides a structured perspective with 14 domains, systematically describing the determinants of behavior (refer to Table S2 in Multimedia Appendix 1 for its structure). This step enables a more precise and context-specific understanding of the obstacles and facilitators. Finally, to convert these insights into actionable strategies, the identified TDF domains were linked to specific BCTs through the established mapping method [21,34], leveraging the hierarchical BCT taxonomy (version 1; refer to Table S3 in Multimedia Appendix 1).

#### Priority Assessment of BCTs

To guide practical implementation, we evaluated the feasibility of the identified BCTs using the Affordability, Practicability, Effectiveness and Cost-Effectiveness, Acceptability, Side-effects or Safety, and Equity criteria [35]. This process involved independent ratings by 2 researchers on 6 dimensions (affordability, practicality, effectiveness, acceptability, side effects, and equity), based on empirical evidence and contextual knowledge. Discrepancies were resolved through discussion with a third expert. Each BCT was subsequently classified as having high, medium, or low feasibility. The specific definitions and classification rules are detailed in Tables S4 and S5 in Multimedia Appendix 1. This feasibility rating was combined with the target level of each BCT (individual, provider, or system).

## Results

### Literature Search

**Figure 1.**
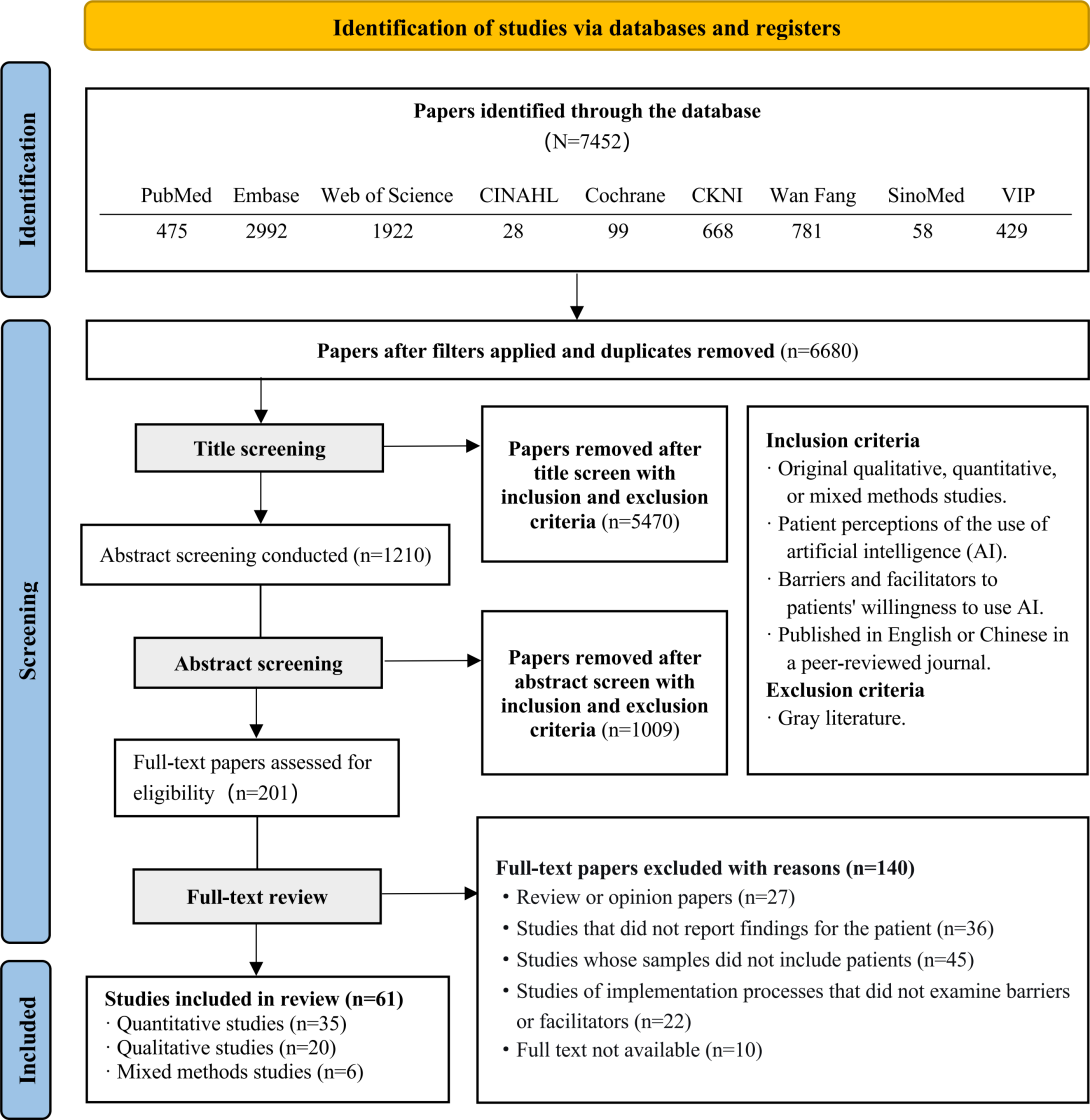
Flow diagram for systematic review.

A comprehensive search of 9 electronic databases using a refined search strategy identified 7452 records. After removing duplicates, 6680 records remained. Following an initial screening of titles and abstracts, 128 records were deemed relevant. Subsequently, full-text screening was performed according to the inclusion and exclusion criteria, excluding irrelevant papers. Ultimately, 61 studies were included in the final review. All literature was used to extract barriers and facilitators, with 53 studies used to extract potential interventions. The literature screening process is shown in Figure 1.

### Study and Patient-Reported Experience Measure Description

This study included 61 studies categorized by research design: 20 qualitative (primarily semistructured interviews), 2 randomized controlled trials, 6 mixed methods, 6 quantitative descriptive, and 27 nonrandomized studies. Quantitative studies mainly used questionnaires, with one adapting the UTAUT2 model [36]. Sample sizes ranged from 9 to 2899 participants. Geographically, studies spanned 19 countries, with 45 from 15 developed countries (eg, 15 from the United States) and 16 from 4 developing countries (eg, 9 from China). Detailed characteristics of the included studies and participants are summarized in Table 2.

**Table 2.** Features of the studies and participants included, stratified by study design.

Authors, country, year	Study type (quality score)	Object of study	Recruitment date	Setting	Aims
Haan et al, Netherlands, 2019 [37]	Qualitative (5)	n=20 (male=11) Age: no restriction Health status: no restriction	2018/07‐2018/08	Radiology of the University Medical Center Groningen	To develop an understanding of patients’ level of knowledge of AI^a^ and to explore the meanings patients ascribe to key topics.
Jalil et al, Australia, 2019 [38]	Qualitative (5)	n=9 (male=5) Age: no restriction Health status: T2D^c^ for at least 12 months	None	None	To investigate the UX^b^ of patients with T2D using a telehealth in-home monitoring device to manage T2D from home.
Adams et al, Canada, 2020 [39]	Qualitative (5)	n=17 (male=6) Age: no restriction Health status: no restriction	None	None	To explore patients’ initial perceptions of AI in the context of radiology and how AI tools can be developed and evaluated in ways that are consistent with patients’ values.
Nelson et al, United States, 2020 [40]	Qualitative (5)	n=48 (male=22) Age: ≥18 years Health status: history of melanoma, history of nonmelanoma skin cancer only, and no history of skin cancer	2019/05‐2019/08	Brigham and Women’s Hospital and the melanoma clinics at the Dana-Farber Cancer Institute	To explore how patients conceptualize AI and perceive the use of AI for skin cancer screening.
Benrimoh et al, Canada, 2021 [41]	Qualitative (5)	n=20 (male=13） Age: no restriction Health status: no restriction	None	Steinberg Center for Simulation and Interactive Learning	To explore the use of a simulation center environment in evaluating the usability of Aifred.
Musbahin et al, United Kingdom, 2021 [42]	Qualitative (5)	n=28 (male=11) Age: ≥18 years Health status: no restriction	None	University patient involvement mailing list	To address this knowledge gap by applying the nominal group technique to explore patient public views on AI.
Zhang et al, United States, 2021 [43]	Qualitative (5)	n=13 (male=9) Age: no restriction Health status: no restriction	2019/08‐2019/09	University Institutional Review Board	To understand patients’ perceptions and acceptance of using AI technology to interpret their radiology reports.
Mikkelsen et al, Denmark, 2023 [44]	Qualitative (5)	n=10 (male=6) Age: ≥18 years Health status: no restriction	2019/10‐2022/01	Denmark health care system	To uncover patient perspectives on trust regarding the patient GP^d^ relationship.
Pelayo et al, United States, 2023 [45]	Qualitative (5)	n=20 (male=8) Age: ≥18 years Health status: diagnosed with type 1 or type 2 diabetes	2022/07‐2022/11	Federally Qualified Health Center	To understand the perspectives of Latin patients with diabetes on ophthalmology, AI-based image interpretation, and general virtual care to prevent avoidable blindness in this population.
Pelly et al, Australia, 2023 [46]	Qualitative (5)	n=38 (male=14) Age: ≥18 years Health status: people with a history of myocardial infarction	2020/11‐2021/12	Cardiac Health Center	To explore the opinions of PHMI^e^ and health professionals on the use of AI for secondary prevention of MI^f^.
Robertson et al, United States, 2023 [47]	Qualitative (5)	n=41 (male=20) Age: ≥18 years Health status: patients with type 1 or type 2 diabetes	None	None	To support and ensure safe use of AI or ML^g^ technologies in health care.
Neves et al, China, 2023 [48]	Qualitative (5)	n=12 (male=15) Age: ≥18 years Health status: patients with stroke confirmed by the Chinese guidelines for the prevention	2021/06‐2022/03	Neurology department of a general hospital	To investigate the experience of robot-assisted gait training experience in patients with stroke.
Godoy Junior et al, Italy, 2024 [49]	Qualitative (5)	n=27 (male=16) Age: no restriction Health status: adult persons with Parkinson disease with a minimum of 2 years with PD^i^ diagnosis	2021/09‐2022/08	None	To explore patient and neurologist perspectives on AI-assisted RMS^h^.
Maris et al, Germany, 2024 [50]	Qualitative (5)	n=24 (male=10) Age: no restriction Health status: those who had either an ICD^j^ and/or a heart condition with increased risk of SCD^k^	None	Dutch ICD Patient Association	To explore perspectives of patients on the ethical use of AI, particularly in clinical decision-making regarding the implantation of an ICD.
Sachdeva et al, Cameroon, 2024 [51]	Qualitative (5)	n=32 (male=8) Age: 30‐49 years Health status: who had previously participated in the CC^l^ screening program	2022/08	DSchang Regional Annex Hospital	To explore the acceptability and perspectives of women in DSchang regarding the usage of a screening tool for CC relying on AI.
Tursynbek et al, Philippines, 2024 [52]	Qualitative (5)	n=30 (male=10) Age: no restriction Health status: no restriction	2024/02‐2024/03	Westhead Hospital	To describe patients’ perceptions of medical AI and its integration into the health care system in Kazakhstan.
Gundlack et al, Germany, 2025 a [53]	Qualitative (5)	n=35 (male=22) Age: no restriction Health status: no restriction	2022/06‐2023/03	A district hospital for psychological illnesses	To explore how patients perceive AI in medical care, focusing on relationships with physicians and ethical aspects.
Gundlack et al, Germany, 2025 b [12]	Qualitative (5)	n=35 (male=22) Age: no restriction Health status: no restriction	2022/06‐2023/03	Martin Luther University Halle-Wittenberg	To explore perceptions toward acceptance, challenges of implementation, and potential applications of AI in medical care.
Krisnan et al, Malaysia, 2025 [13]	Qualitative (5)	n=17 (male=14) Age: no restriction Health status: no restriction	2023/08	Hospital Tengku Ampuan Rahimah	To examine patient perceptions and attitudes toward the use of AI in diabetes care.
Trivedi et al, Australia, 2025 [54]	Qualitative (5)	n=30 (male=15) Age: no restriction Health status: no restriction	2021/08‐2022/05	Westhead Hospital	To explore patient perspectives on receiving support from a conversational AI support program.
Sebastian et al, United States, 2023 [55]	RCT (3)	n=150 (male=68) Age: no restriction Health status: no restriction	None	None	To examine whether communication strategies are more successful in overcoming factors that hinder AI product adoption among patients.
Zhou et al, China, 2022 [56]	RCT (5)	n=776 (male=39) Age: no restriction Health status: no restriction	None	None	To explore the influence of the degree of AI involvement on patients’ WTA^m^ and to reveal whether the reason is caused by patients’ perceived threat to outside groups.
Yang et al, China, 2019 [57]	Cross-sectional (4)	n=527 (male=199) Age: ≥18 years Health status: Chinese patients with cancer, who were informed of their cancer diagnosis	None	Cancer Center of Sichuan University	To assess the attitudes of Chinese patients with cancer toward the clinical use of AIM^n^, and to analyze the possible influencing factors.
Jutzi et al, Germany, 2020 [58]	Cross-sectional (4)	n=298 (male=80) Age: no restriction Health status: no restriction	2019/11‐2020/01	Dermatological University Hospital	To evaluate the patients’ view of AI in melanoma diagnostics in Germany, with a particular focus on patients with a history of melanoma.
Meyer et al, United States, 2020 [59]	Cross-sectional (3)	n=329 (male=74) Age: no restriction Health status: no restriction	2018/03‐2018/05	The Isabel Symptom Checker	To examine patients’ experiences using an AI-assisted online symptom checker.
Ongena et al, Netherlands, 2020 [60]	Cross-sectional (5)	n=155 (male=86) Age: ≥18 years Health status: no restriction	2018/11‐2019/03	University Medical Center Groningen	To develop and validate a standardized patient questionnaire on the implementation of AI in radiology.
Aggarwal et al, United Kingdom, 2021 [61]	Cross-sectional (5)	n=408 (male=173) Age: ≥16 years Health status: no restriction	2018/06‐2018/09	University Teaching Hospital	To identify current awareness regarding health data research, and to obtain their opinions and views on data sharing for AI research purposes.
Esmaeilzadeh et al, United States, 2021 [62]	Cross-sectional (5)	n=634 (male=355) Age: no restriction Health status: no restriction	2022/05	Institutional Review Board of Florida International University	To examine how potential users (patients) perceive the benefits, risks, and use of AI clinical applications for their health care purposes.
Liu et al, China, 2021 [63]	Cross-sectional (2)	n=428 (male=206) Age: 18‐85 years Health status: no restriction	None	First Affiliated Hospital of Jinan University	To visualize and measure patients’ heterogeneous preferences from various angles of AI diagnosis.
Lennartz et al, Germany, 2021 [64]	Cross-sectional (3)	n=229 (male=99) Age: no restriction Health status: no restriction	2020/01‐2020/05	Institute for Diagnostic and Intervention Radiology	To investigate patients’ opinions on the use of AI in different aspects of the medical workflow.
Armero et al, United States, 2022 [65]	Cross-sectional (4)	n=349 (male=176) Age: ≥18 years Health status: enrolled patients presenting to the labor and delivery unit at a tertiary care	2019/11‐2020/06	Labor and Delivery Unit of Brigham and Women’s Hospital	To evaluate and understand pregnant patients’ perspectives on the implementation of AI in clinical care.
Yulan et al, China, 2022 [66]	Cross-sectional (5)	n=446 (male=244) Age: no restriction Health status: not target-specific disease populations	2020/02‐2020/04	West China Hospital of Sichuan University	To understand older patients’ willingness to use AI robots and the influencing factors.
Khullar et al, United States, 2022 [67]	Cross-sectional (4)	n=926 (male=455) Age: no restriction Health status: no restriction	2019/12	None	To understand public perceptions of the use of AI in diagnosis and treatment.
Kosan et al, Germany, 2022 [68]	Cross-sectional (5)	n=140 (male=69) Age: ≥18 years Health status: no restriction	2021/02‐2021/03	Charité–Universitätsmedizin Berlin Dental Clinic	To assess patients’ perspectives on AI in dentistry scientifically for radiography caries detection, and the impact of AI-based diagnosis on patients’ trust.
Ayad et al, Germany, 2023 [9]	Cross-sectional (4)	n=330 (male=165) Age: ≥18 years Health status: no restriction	2021/12‐2022/03	University Hospital Munster	To assess patients’ knowledge and perceptions of AI in dentistry.
Kawsar et al, United Kingdom, 2023 [69]	Cross-sectional (3)	n=268 (male=114) Age: ≥18 years Health status: at least one skin lesion that could be photographed	2020/02‐2021/08	Chelsea and Westminster Hospital	To explore patients’ perspectives on the use of AI as part of their skin cancer management pathway.
Mahlknecht et al, Italy, 2023 [70]	Cross-sectional (4)	n=116 (male=52) Age: no restriction Health status: no restriction	2021/09‐2021/11	The Institute of General Practice and Public Health in Bolzano	To evaluate both patients’ and physicians’ attitudes toward these tools in Italian general practice settings.
Parry et al, United States, 2023 [71]	Cross-sectional (4)	n=397 (male=141) Age: ≥18 years Health status: no restriction	2020/03‐2021/08	A large urban academic center and a rural health system.	To investigate the patient opinion on the use of AI in orthopedics.
Temple et al, United Kingdom, 2023 [72]	Cross-sectional (5)	n=95 (male=68) Age: no restriction Health status: no restriction	2021/11‐2022/03	A specialist UK cancer center.	To adapt and use a validated patient questionnaire to develop an understanding of current patient views on the use of AI in radiotherapy.
Baghdadi et al, Saudi Arabia, 2024 [73]	Cross-sectional (5)	n=382 (male=109) Age: ≥18 years Health status: no restriction	2022/07‐2022/12	Tertiary care hospital	To examine patients’ attitudes toward the use of AI as a tool in diagnostic radiology.
Bahadir et al, Turkey, 2024 [74]	Cross-sectional (5)	n=272 (male=107) Age: ≥18 years Health status: advanced occlusal caries	2023/04‐2024/01	The clinic of restorative dentistry	To evaluate patients’ attitudes toward the use of AI in dental radiographic detection.
Esin et al, Turkey, 2024 [75]	Cross-sectional (4)	n=725 (male=319) Age: ≥18 years Health status: undergone surgery	2021/05‐2021/06	Tepee Training and Research Hospital	To evaluate the opinions of individuals regarding the use of AI and robots in the field of health care.
Fransen et al, Western European, 2024 [76]	Cross-sectional (5)	n=212 Age: no restriction Health status: patients suspicious with prostate cancer undergoing prostate MRI^o^	2023/01‐2023/10	At 3 Western European medical institutes	To investigate patients’ acceptance of AI for diagnosing prostate cancer on MRI scans and the factors that influenced their trust in AI diagnoses.
Huang et al, Singapore, 2024 [36]	Cross-sectional (5)	n=100 (male=56) Age: no restriction Health status: no restriction	2021/09‐2022/06	National Heart Center	To better understand factors influencing patient usage intention.
Riedl et al, Germany, 2024 [77]	Cross-sectional (5)	n=1183 (male=535) Age: ≥18 years Health status: no restriction	2022	A market research company	To investigate patients’ preferences for interacting with a human doctor or an AI system.
Alsanosi et al, Saudi Arabia, 2025 [11]	Cross-sectional (5)	n=385 (male=154) Age: no restriction Health status: no restriction	2024/07‐2024/12	Umm Al-Qura University	To explore knowledge, attitudes, and perceptions (KAP) regarding AI in medication adherence among chronic patients in the Makkah region.
Chan et al, Australia, 2025 [78]	Cross-sectional (4)	n=385 (male=211) Age: no restriction Health status: no restriction	2024/06‐2024/10	The Faculty of Medicine and Health	To provide a baseline understanding of patient views about the use of AI/ML in the specific context of radiotherapy to contribute toward future governance of the technology.
Ozcan et al, Texas, 2025 [79]	Cross-sectional (5)	n=518 (male=259) Age: no restriction Health status: no restriction	2023/02‐2023/08	Texas Southwestern Medical Center	To identify the independent factors associated with participants’ acceptance of AI use.
Tirapelli et al, United States, 2025 [80]	Cross-sectional (5)	n=2581 (male=1806) Age: no restriction Health status: no restriction	2023/01‐2024/06	Aarhus University	To evaluate patients’ perceptions of the use of AI in dental imaging diagnostics across 6 centers worldwide.
Xiangde, China, 2019 [81]	Quantitative descriptive (1)	n=446 (male=236) Age: no restriction Health status: conscious outpatient and inpatient patients	2018/05‐2018/06	The Third Hospital of Chongqing	To understand patients’ cognition and trust in AI health care, and analyze the reasons.
Menghan and Weihua, China, 2019 [82]	Quantitative descriptive (4)	n=550 (male=271) Age: no restriction Health status: no restriction	None	Different hospitals in Guangdong province	To study the influence mechanism of the use of hospital intelligent medical systems on patients’ satisfaction with medical treatment.
Siwen and Jiudi, China, 2022 [83]	Quantitative descriptive (3)	n=200 (male=117) Age: ≥18 years Health status: esophageal cancer staging ranges from stage I to stage IV	2019/07‐2020/12	Sun Yat Sen University Cancer Center	To investigate the acceptance of the P2 robot by patients, families, and medical staff for health education in patients with esophageal cancer surgery, propose targeted improvement measures, and provide a basis for the future widespread use of robots in health education.
Jin et al, China, 2023 [84]	Quantitative descriptive (5)	n=39 (male=20) Age: no restriction Health status: meets the diagnostic criteria for dysphagia	2022/03‐2022/05	Department of Neurology, Beijing Chaoyang Hospital	To design and develop a swallowing function training system suitable for patients with stroke with swallowing disorders.
Haggenmüller et al, United States, 2024 [85]	Quantitative descriptive (5)	n=178 (male=89) Age: no restriction Health status: no restriction	None	American Academy of Dermatology	To investigate the criteria required for patients and dermatologists to accept AI-systems.
Macri et al, Australia, 2024 [86]	Quantitative descriptive (4)	n=15 (male=3) Age: ≥18 years Health status: vitreoretinal surgery, in which postoperative face-down positioning is used	2022/12‐2023/09	The Royal Adelaide Hospital	To examine the acceptability of an AI-generated presenter in a patient information video about face-down positioning after theoretical surgery.
Palmisciano et al, United Kingdom, 2020 [87]	Mixed methods (5)	n=20+107 (male=45) Age: no restriction Health status: no restriction	Qualitative: 2019/09 and quantitative: 2019/10	Department of Neurosurgery	To evaluate attitudes of patients and their relatives regarding the use of AI in neurosurgery.
van der Zander et al, Netherlands, 2022 [88]	Mixed methods (3)	n=377 (male=155) Age: ≥18 years Health status: who underwent an endoscopic procedure	2020/04‐2021/08	Catharina Hospital Eindhoven	To investigate the perspectives on AI in health care among patients with GI^p^ disorders.
Katirai et al, Japan, 2023 [89]	Mixed methods (5)	n=11 (male=5) Age: no restriction Health status: no restriction	2021/02‐2021/04	The Osaka University Graduate School of Medicine	To provide a snapshot of patient and public perceptions of AI in health care in the Japanese context.
Robertson et al, United States, 2023 [47]	Mixed methods (5)	n=24+2875 (male=1092) Age: no restriction Health status: no restriction	Qualitative: 2020/02-2020/12 and quantitative: 2021/01‐2021/02	Clinics in Tucson, Arizona	To determine how diverse patient populations feel about the use of AI diagnostic tools.
Gonzalez et al, United States, 2024 [90]	Mixed methods (5)	n=281 (male=140) Age: ≥18 years Health status: no restriction	Phase I: 2023/06 and Phase II: 2023/07‐2023/10	Academic medical center in St. Louis	To assess public and surgical patient attitudes and perspectives on ML-CDSS^q^ use in perioperative care.
Witkowski et al, United States, 2024 [91]	Mixed methods (4)	n=600 (male=292) Age: ≥18 years Health status: no restriction	2023/08	A leading market research provider	To explore the extent to which patients are confident in and comfortable with the use of these technologies when it comes to their own individual care, and identify areas.

a AI: artificial intelligence.

b UX: user experience.

c T2D: type 2 diabetes.

d GP: general practitioner.

e PHMI: people with a history of myocardial infarction.

f MI: myocardial infarction.

g ML: machine learning.

h RMS: remote monitoring system.

i PD: Parkinson disease.

j ICD: implantable cardioverter-defibrillator.

k SCD: sudden cardiac death.

l CC: cervical cancer.

m WTA: willingness to accept.

n AIM: artificial intelligence in medicine.

o MRI: magnetic resonance imaging.

p GI: gastrointestinal.

q ML-CDSS: machine learning–clinical decision support system.

The study population includes patients using medical AI aged ≥18 years, recruited from communities, hospitals, and online surveys. Patient types varied, with a focus on patients with cancer (n=11), radiology (n=8), perioperative (n=4), diabetes (n=3), dental (n=5), and stroke (n=2). Nine studies included patient and health care professional perspectives; only patient data were analyzed to align with research objectives. The comprehensive data and full extraction details are provided in Table S6 in Multimedia Appendix 1 due to space constraints.

### Quality Assessment

According to the MMAT evaluation, the average score of the included studies was 4.4. Approximately 85% (n=53) of the studies met all or 80% of the quality assessment criteria. About 11% (n=6) of the studies met 60% of the quality assessment criteria. Two studies met only 40% or less of the quality assessment criteria. In general, the methodological quality of the included studies was assessed as relatively high. The MMAT User Manual mentions the treatment of low-quality studies, which are rounded off according to the topic of the study; in this study, these 2 papers were valuable in contributing to the research topic and were therefore not excluded [25]. The MMAT scores for each included study are provided in Table 2, and detailed item-level assessments are provided in Table S7 in Multimedia Appendix 1.

### Identified Barriers and Facilitators

#### Overview

**Figure 2.**
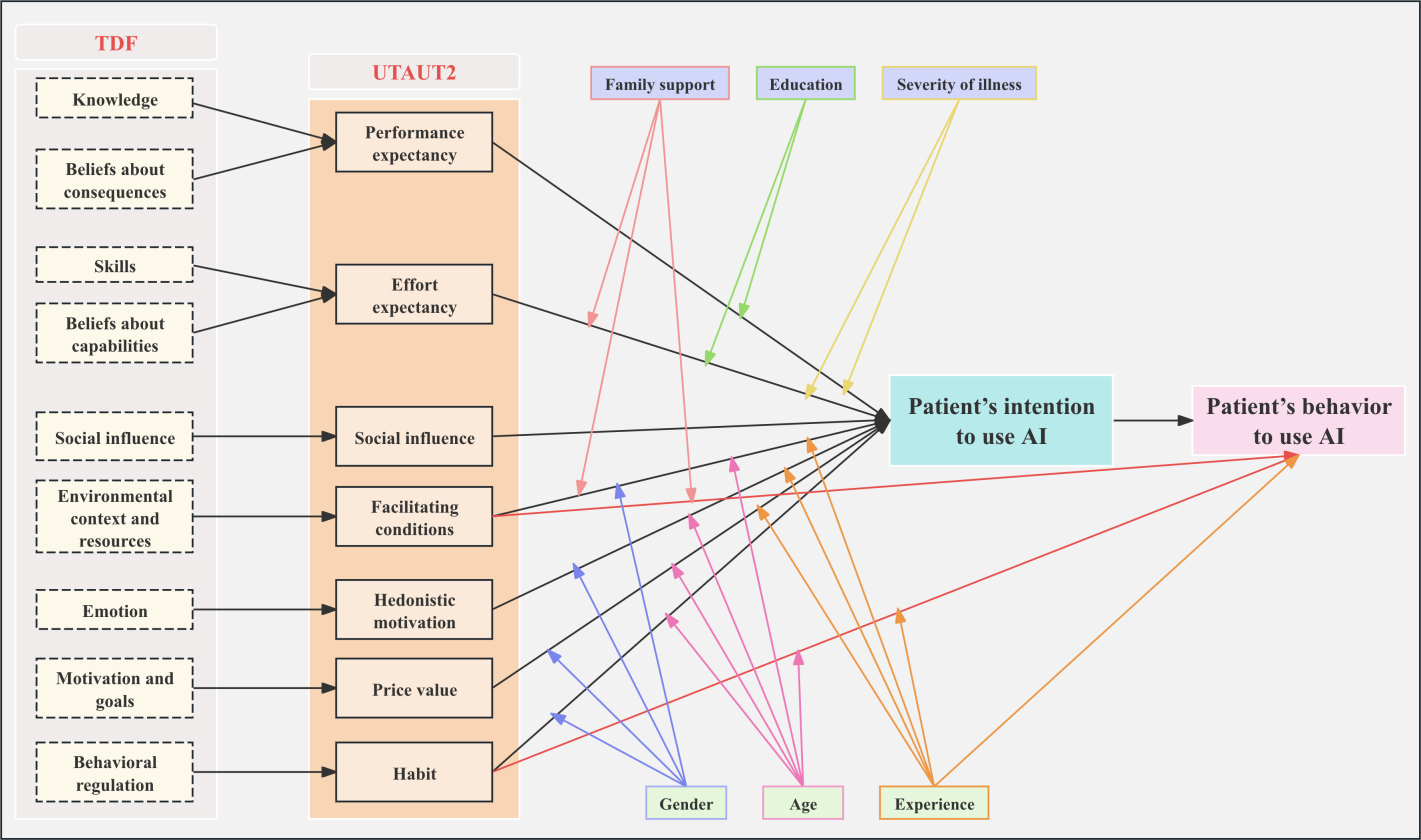
An integrated model of patient acceptance of artificial intelligence (AI) in health care: combining Theoretical Domains Framework (TDF) and Unified Theory of Acceptance and Use of Technology 2 (UTAUT2) framework.

To ensure transparency in the analytic process, we followed structured steps. First, 2 researchers independently conducted open coding and reached a consensus through discussion, forming an initial codebook. These codes were then categorized into the 7 core dimensions of UTAUT2 (eg, “concern about effectiveness” was classified under “performance expectancy”). Subsequently, codes within each UTAUT2 dimension were mapped to specific TDF domains to explain their underlying behavior change mechanisms (eg, “public misunderstanding” corresponded to the “knowledge” domain). The model correspondence is shown in Figure 2. When overlaps in mapping occurred, the primary corresponding domain was determined through team discussion based on the most direct and dominant driving mechanism reflected in the data. Ultimately, UTAUT2 provided a clear structure of acceptance dimensions (the “what”), while TDF offered an in-depth explanation of behavioral mechanisms (the “why”), with their integration jointly supporting the presentation of the results (as shown in Table 3). Performance expectancy and effort expectancy were the most cited barriers or facilitators to patients’ use of AI in medicine. The corresponding text describing barriers and facilitators is provided in Table S8 in Multimedia Appendix 1.

**Table 3.** Barriers and facilitators to patient acceptance of medical artificial intelligence (AI)^a^, mapped to the integrated Unified Theory of Acceptance and Use of Technology 2 (UTAUT2) and Theoretical Domains Framework (TDF).

UTAUT2^b^ model construct and TDF^c^ model corresponding domain	Barriers	Facilitators
Performance expectancy		
Knowledge	Public prejudice and misunderstanding of new technology Public fear and resistance to new technology	Understanding the importance of AI^a^ in health care
Beliefs about consequences	Possibly worse outcomes Lack of trust in AI^a^ Difficult to measure effectiveness	Belief in AI’s faster diagnostic speed, high efficiency, and accurate diagnostic results
Effort expectancy		
Skills	Complex technology operation Need to invest more time in learning and adapting to new technology applications and operational processes	Convenient operation
Beliefs about capabilities	Uncertainty about new technology Reduced self-efficacy	Assisting the doctor’s decision-making function Enhanced self-efficacy
Social influence		
Social influences	Cultural adaptability and intergroup threat Few people around or family members use AI Unclear medical responsibility attribution Insufficient patient-doctor communication	Public trust Recommendations from trusted sources (doctors) Improved patient-doctor communication
Facilitating conditions		
Environmental context and resources	Insufficient patient privacy security protection Cybersecurity not ensured Insufficient data reliability and transparency Technical errors and false information obtained Lack of humanistic care in AI Imperfect AI technology	Protection of patient privacy (sensitive issues) Cybersecurity ensured Reliable data (fairness, interpretability, and transparency)
Hedonistic motivation		
Emotion	Poor new technology usage experience Lack of personalized experience	Novel experience
Price value		
Motivation and goals	High cost of new technology application Expensive (unequal opportunities) Fear of high trial-and-error costs	Cost savings
Habit		
Behavioral regulation	Unaccustomed to using AI technology May make patients unwilling to persist in using new medical technology to manage their health.	—^d^

a AI: artificial intelligence.

b UTAUT2: Unified Theory of Acceptance and Use of Technology 2.

c TDF: Theoretical Domains Framework.

d Not available.

#### Performance Expectancy

Performance expectancy is the extent to which patients feel that the use of AI has contributed to the perception of illness [14], which can be further explained by the dimensions of knowledge, beliefs about outcomes, behavioral regulation, and environmental context and resources in TDF. In summary, patients’ performance expectancy of medical AI is primarily shaped by a tension between widespread cognitive biases and misconceptions (n=28) and a strong belief in its efficacy when supported by empirical evidence (n=31).

#### Effort Expectancy

Effort expectancy reflects the effort required for patients to use AI, linked to TDF dimensions such as skills, cognition, and behavioral regulation [14]. The foremost challenges are high operational complexity and substantial learning investment (n=17), whereas facilitation hinges on interface optimization and process simplification to reduce cognitive load (n=22). This highlights that ease of use is not merely a convenience but a critical prerequisite for patient engagement with AI tools.

#### Social Influence

Social influence, defined as the impact of surrounding groups on AI adoption, includes subjective norms and public trust [14]. Low technology adoption, reduced clinician-patient interactions (n=26), and ethical concerns (n=27) were frequently reported as barriers. In contrast, facilitators such as endorsements from authoritative institutions and enhanced clinician-patient communication (n=9) were noted as promoting acceptance. Collectively, these findings indicate that social and relational factors, including interpersonal trust and perceived legitimacy, are consistently salient in shaping patient responses to AI. Within the TDF framework, this theme aligns with domains of interpersonal relationships and professional roles.

#### Facilitating Conditions

Facilitating conditions reflect organizational support for system use, aligning with the TDF dimension of environmental context and resources [14]. Key barriers included data reliability issues, privacy vulnerabilities, and cybersecurity risks, with insufficient privacy protection being a significant concern (n=22). Facilitators focused on establishing transparent data governance, enhancing privacy encryption, and reinforcing cybersecurity protocols to ensure fairness, interpretability, and transparency (n=12).

#### Hedonistic Motivation

This refers to the degree of pleasure or enjoyment an individual derives from using new technology [15], corresponding to the TDF dimensions of emotion, affect, motivation, and goals. The most frequently reported barrier was the mechanized nature of technology use, which led to a lack of empathy, increased patient anxiety, and a reduced sense of self-efficacy. In contrast, the primary facilitator was the design of personalized interaction models to enhance positive user experiences and integrate emotion-aware algorithms to mitigate patients’ negative psychological responses.

#### Habit

Habit is the tendency to automate behaviors resulting from the patient’s frequent use of technology [92]. The corresponding TDF dimension is behavioral regulation. Patients’ reluctance to adopt new technologies was the most frequently reported barrier due to their preference for traditional methods.

#### Price Value

Price value reflects the balance between technology benefits and economic costs [15,93], linked to the TDF domain of environmental context and resources. A cost-benefit calculus shapes perceptions of price value. The primary barrier is the high upfront financial cost and associated risk (n=14), while facilitators focus on policy subsidies and training to alleviate economic burdens and demonstrate long-term value (n=10). This economic perspective confirms that cost-effectiveness and affordability are practical, decisive factors for many patients.

#### Moderating Variables

The UTAUT2 model highlights moderating variables (eg, age, gender, and experience) influencing AI adoption in health care. This study expanded the analysis to include disease severity and education level. Experience, defined as perceived performance enhancement, was linked to TDF dimensions such as knowledge and environmental context. Prior experience increased AI acceptance, while gender and age did not significantly impact most studies [47,71]. Although findings varied, disease severity and education level were identified as additional moderators. Further research is needed to clarify these relationships and develop targeted interventions for AI adoption.

### BCTs and Implementation Strategies

Through group discussions, 27 factors from UTAUT2 and TDF domains were mapped to 93 potential BCTs. After consolidation, 25 distinct BCTs were assessed using the Affordability, Practicability, Effectiveness and Cost-Effectiveness, Acceptability, Side-effects or Safety, and Equity criteria and categorized by feasibility: 6 (24%) as high, 14 (56%) as medium, and 5 (20%) as low (detailed in Table S10 in Multimedia Appendix 1). High-feasibility BCTs (eg, 4.1 and 5.1) are low cost, easy to integrate, and well accepted. Medium-feasibility BCTs (eg, 6.1 and 15.1) are evidence-based but face operational constraints, while low-feasibility BCTs (eg, 9.1 and 12.1) require system- or policy-level changes beyond clinical teams. Concrete examples for these BCTs were drawn from the literature (Table S9 in Multimedia Appendix 1), resulting in 40 targeted interventions. Complete results are shown in Table 4 (the serial numbers and other details of the relevant BCT are provided in Table S3 in Multimedia Appendix 1).

**Table 4.** Behavior change techniques (BCTs) mapped from identified barriers and facilitators to inform interventions for enhancing patient artificial intelligence (AI) acceptance.

UTAUT2^a^ component and relevant BCT^b^	Target level	Feasibility	Example quotes
Performance expectancy			
TDF^c^ domain: knowledge			
Instructions on how to perform the behavior	Patient	High	Provide information on the effects of using AIM^d^ and educating patients about the benefit of the current health care system [76], and participants requested that information and training be provided in a number of different ways (eg, pamphlets, in-person training, computer-guided supports, and sharing of patient experiences) [94].
Demonstration of the behavior	Patient	High	Detailed instructional content: provide a comprehensive, step-by-step guide on the functionality and operation of AI^e^ systems, including visual aids such as diagrams, flowcharts, and videos to illustrate key concepts and procedures.
TDF domain: beliefs about consequences			
Information about health consequences	Patient	High	Provide information (eg, written, verbal, and visual) about AI; educate and involve patients in the future direction of this technology; demonstrating to patients that the use of AI in their own health care is safe, effective, and ethical; and establish a baseline educational level [72].
Salience of consequences	Patient	Medium	Using methods specifically designed to emphasize the consequences of performing the behavior to make them more memorable.
Anticipated regret	Patient	Medium	Enhance patients’ awareness of the usefulness of AI by showcasing successful cases to patients, provide them with a preliminary understanding of the expected outcomes.
Effort expectancy			
TDF domain: skills (constructs other than interpersonal skills)			
Goal setting (behavior)	Patient	High	Devise ways to perform the behavior: provide participants with an AI device; patients need to understand all information about AI and require that all health care providers undergo technology training [47].
Feedback on behavior	Patient	Medium	Monitor and provide feedback on exercise performance (exercise type, frequency, duration, and intensity), and monitor and show performance via mobile phone or sports bracelet.
Self-monitoring of behavior	Patient	High	Establish a method for participants to monitor and record their AI use.
Feedback on outcomes of behavior	Patient	Medium	Provide feedback about body metrics using AI devices: use sport bracelets, heart rate bands, and other devices to monitor physiological indicators; by optimizing the structure of the application and designing user-friendly apps with simple navigation and clear interfaces, usability can be improved, thereby increasing patient acceptance [84].
TDF domain: beliefs about capabilities			
Verbal persuasion about capability	Clinician	Medium	Enhance the professional quality of medical staff by improving the professional capabilities of medical staff, enhancing patients’ trust in the medical staff and the AI products they recommend.
Social influences			
TDF domain: social influences			
Social support (unspecified）	All	Low	Social support (practical) formulates comprehensive laws and regulations; clarifies the liability attribution of AI in the medical field to protect the legal rights and interests of patients and medical staff [63]; strengthens publicity efforts, establishes management systems and standards, and reinforces communication and guidance.
Information about others’ approval	Patient	Medium	Provide information about what other people think about AI; make participants aware that many patients have a positive attitude toward AI; and leverage primary care providers’ recommendations [45].
Restructuring the social environment	All	Medium	Provide participants with a social environment suitable for using AI; change perspectives of participants’ family members so that they support the participant’s choice; emphasize communication between patients and medical staff. When adopting AI technology, focus on patient experience, reduce repetitive work time of medical staff, increase communication with patients, and implement the core concept of humanistic care nursing.
Facilitating conditions			
TDF domain: environmental context and resources			
Monitoring of behavior by others without feedback	Clinician	Low	Monitoring of participants’ use without feedback: monitoring of participants’ use by professionals (eg, physical therapists, nursing staff, and so on) to optimize the delivery of follow-up interventions.
Credible source	System	Low	Ensure reliable and transparent data sources: safeguard the security of the network environment used by AI and ensure that patients’ privacy data is not leaked.
Restructuring the physical environment	System	Low	Provide a safe and reliable data environment; formulate relevant policies and behavioral norms to ensure data security and reliability. Provide a safe place to use AI; ensure protection of data and provide patients with assurance regarding strict conditionality. Convey purpose, benefits, and potential risks in written form (brochures) for patients [51]; ensure suitable AI equipment and convenient access; protect patient privacy and increase transparency in medical data processing; enhance transparency in the decision-making process [65].
Restructuring the social environment	System	Medium	Provide a social environment that promotes AI use, ensuring AI tools preserve the integrity of the human physician-patient relationship [40].
Adding objects to the environment	System	Low	Add objects to the environment to facilitate behavior, provide personalized AIM services based on patients’ specific situations to enhance their perceived value.
Hedonistic motivation			
TDF domain: emotion			
Monitoring of emotional consequences	Clinician	Medium	Provide emotional support (from professionals, exercise partners, family, or friends)
Information about emotional consequences	Clinician	Medium	Monitoring participants’ feelings after use: monitor participants’ feelings to avoid negative experiences and adjust subsequent tasks accordingly.
Price value			
TDF domain: motivation and goals			
Review outcome goals	All	Medium	Empowering personalized care and patient autonomy with feedback: AI assists health care professionals in providing personalized care while safeguarding patient autonomy and decision-making ability. Meanwhile, patient usage feedback is promptly adopted to optimize AI use and enhance patient satisfaction.
Habit			
TDF domain: behavioral regulation			
Goal setting (behavior)	Patient	High	Goal setting: encourage patients to gradually adapt to AI operation through exploration and practice.
Action planning	System	Low	Tailored using programs for participants: regulatory agencies should establish normative standards and evaluation guidelines for implementing AI in health care in cooperation with health care institutions [62].
Feedback on behavior	Patient	Medium	Monitor and provide feedback on exercise performance (eg, exercise type, frequency, duration, and intensity) via mobile phone or sports bracelet.
Habit formation	Clinician	Medium	Timely adoption of patient usage feedback: regularly follow up with patients to understand their usage of AI technology and willingness to continue using it.

a UTAUT2: Unified Theory of Acceptance and Use of Technology 2.

b BCT: behavior change technique.

c TDF: Theoretical Domains Framework.

d AIM: artificial intelligence in medicine.

e AI: artificial intelligence.

## Discussion

### Principal Findings

This review aimed to explore the main barriers and enablers underlying patients’ adoption of AI in hospital settings. Prior studies have examined the intention to use AI among different types of patients, but there has been no systematic synthesis of barriers and facilitators. This study used an innovative approach to synthesize patients’ views on using AI in medicine, integrating barriers and facilitators of patient adoption of AI in medicine based on the UTAUT2 and TDF.

Our findings reveal that AI in medicine adoption is driven by a 3-tier mechanism: “technology acceptance, contextual modulation, and ethical security.” Moreover, individual differences influence AI acceptance. For example, age moderates the perception of technological complexity, with older adults favoring simpler systems [67,72]. Education level is a cognitive mediator, affecting performance expectancy by shaping AI-related knowledge [51,60,65,71,75,84]. Additionally, disease severity exhibits an inverted U-shaped relationship with AI acceptance, where patients with moderate conditions demonstrate the highest acceptance [64,73].

Regarding the most important barriers and enablers identified in this review, performance expectancy and effort expectancy were the most cited factors influencing patients’ use of AI in medicine. This finding substantiates the results of earlier studies [37,61,67]. Patients’ recognition of AI’s diagnostic efficiency and accuracy emerges as the primary determinant of acceptance under performance expectancy [15,30], aligning with findings from Schmitz et al [16] in cross-national telemedicine research. Additionally, within the TDF framework, the “knowledge” domain significantly impacts patient adoption of AI, highlighting that awareness and understanding of AI technologies are crucial determinants of acceptance and use [37,61,67]. There is a need to improve AI knowledge and increase willingness to use AI, which can be achieved by enhancing patient education through outpatient health seminars and multimedia educational videos.

In addition, the operational feasibility of AI plays a key role in promoting patient use. The perceived value of AI for patients increases significantly when they are able to receive positive feedback and are effectively supported by health care professionals when using it [95]. For example, an AI robotic system that receives positive feedback from health care professionals during rehabilitation training can enhance patients’ confidence and sense of accomplishment, further promoting the usage of AI [96]. However, poorer usability can be a source of fear for patients toward new technologies and, therefore, may hinder their adoption [96]. A “progressive complexity” strategy, embedding educational modules within basic applications, may facilitate gradual comprehension and trust development in AI systems [36,84].

Social networks profoundly shape patient willingness to use AI, extending beyond physician recommendations to include family members, friends, and community influence. Family acceptance acts as a critical multiplier—tech-savvy relatives often facilitate AI adoption by assisting with device operation or interpreting digital health reports, whereas households with low health literacy may resist AI due to distrust of unfamiliar technology [63]. Peer influence also plays a role: patients are more likely to embrace AI-driven tools after observing positive experiences shared by friends or online communities, particularly in managing chronic conditions such as diabetes or hypertension. Conversely, negative anecdotes within social circles (eg, stories of AI misdiagnosis) can amplify skepticism, creating “information cascades” that undermine trust [47]. These dynamics highlight the need for targeted social marketing strategies, for example, family-centered education workshops or peer advocacy programs to address intergenerational and community-specific barriers.

Cultural adaptability is primarily shaped by specific constructs within the UTAUT2 and TDF frameworks, with social influence being the most culturally sensitive dimension. This review finds that in collectivist societies (eg, East Asia), social influence often manifests as deference to family or group consensus [97], effectively shifting the adoption unit from the individual to the collective. Conversely, in individualistic cultures, it leans more on trust in professional authority or empirical evidence. Consequently, implementation strategies must be contextualized: collectivist settings may require designs that facilitate family-inclusive decision-making and leverage community opinion leaders, whereas individualistic contexts should emphasize transparent personal benefits and institutional endorsements. Systematically analyzing culture through these theoretical lenses enables more targeted and effective AI implementation across diverse health care systems.

Ethical safety concerns, particularly the “algorithmic black box,” undermine trust in AI’s reliability among patients and clinicians [45,58,62,64,67]. Explainable AI, by providing transparent diagnostic reasoning and interpretable reports, mitigates skepticism and strengthens technical credibility [39]. While AI may reduce patient embarrassment in sensitive consultations [46], overreliance risks eroding humanistic doctor-patient interactions, exacerbating anxiety [37,40,44,45,49,50]. Balancing privacy protection with human-centered design remains a critical research frontier.

Beyond mitigating ethical and relational barriers, our synthesis points toward the necessity of proactively rehumanizing AI-driven health care. This concept involves intentionally designing AI systems and their implementation contexts to preserve and enhance, rather than replace, essential human elements such as provider presence, empathy, and meaningful interaction. Studies, such as the work of Almokdad et al [98] in digital hospitality, underscore the critical importance of human presence in mitigating the impersonal nature of automated services. Translating this to health care, AI should be positioned as a tool that augments, rather than replaces, the clinician. To operationalize this, concrete strategies include (1) adopting “AI-as-assistant” design paradigms where clinicians remain central in communication and decision-making, (2) implementing empathy-aware algorithms to prompt providers to address patient emotional states, and (3) leveraging AI’s efficiency gains to protect or increase time for direct patient-clinician interaction.

Additional psychological and economic factors include hedonic motivation, habituation, and price-value trade-offs. Positive emotional experiences with AI (eg, intuitive interfaces) boost initial acceptance [36,47,83,89,96], whereas habit-driven preference for traditional methods necessitates proactive demonstration of AI’s clinical advantages [38,62,66,83,87,89]. Although AI adoption may incur higher upfront costs [9,58,63,66,67,81,89], its long-term potential to reduce unnecessary diagnostics and optimize treatment pathways could yield cost savings [88,90]. Future research should contextualize AI’s economic impact within health care settings while prioritizing personalized, culturally sensitive implementations.

The BCT taxonomy (version 1) provides a standardized framework for behavioral interventions in AI implementation [34]. This study advances its practical application by introducing a feasibility lens, which transforms a theoretical list of techniques into a prioritized implementation strategy. Our assessment distinguishes high-feasibility BCTs (eg, instruction and goal setting) as immediately actionable tools for frontline adaptation, such as optimizing interfaces for older adults [99]. Medium-feasibility BCTs, including strategies such as social comparison to address privacy concerns [55], require deliberate planning and resources. Ultimately, sustainable adoption depends on low-feasibility, systemic enablers, such as transparent data governance, which establish the necessary foundation for trust. Integrating barrier mapping with this feasibility-driven prioritization creates a clear, staged model for implementation. It clarifies what clinicians can do now, what requires organizational support, and what must be advocated at the policy level, offering a pragmatic roadmap for integrating AI into health care.

In summary, the primary contribution of this study lies in its theoretical integration and methodological innovation. Distinct from previous research that predominantly focused on technological attributes or isolated psychosocial factors, this review establishes a comprehensive analytical model that links technology acceptance with behavior change by integrating the UTAUT2 and TDF frameworks. Beyond this theoretical contribution, this integrative model deepens the understanding of barriers and facilitators to patient adoption of AI and, importantly, translates these insights into actionable evidence by mapping the findings to specific and prioritized BCTs. This evidence directly informs and empowers multiple stakeholders: it provides health care administrators with a blueprint for implementation strategies, equips clinicians with tools for patient engagement, guides AI developers toward human-centered design principles, and supports policymakers in developing frameworks for ethical evaluation and governance. Ultimately, this review bridges the gap between theoretical understanding and practical application, thereby enhancing the translational value of research on AI acceptance in health care.

### Limitations and Strengths

The strength of this study lies in its systematic synthesis of qualitative and quantitative evidence, conducted through a mixed methods evaluation guided by an integrated theoretical framework. Theoretically, it innovatively combines the UTAUT2 model with the TDF to analyze barriers and facilitators influencing patients’ adoption of AI in health care, thereby providing a dual perspective that incorporates both technology acceptance and behavioral science. This theory-informed approach allows for a coherent interpretation of existing evidence and establishes a robust foundation to support patient-centered AI implementation, while also informing the design of future interventions and health policy initiatives.

This review has several limitations. First, methodological constraints include potential publication bias and language restrictions (Chinese and English only), which may affect the comprehensiveness and cultural generalizability of the findings. Second, the evidence base is limited by the uneven geographical distribution of included studies, with underrepresentation from developing countries, and the retention of 2 lower-quality studies that did not alter the overall conclusions. Third, the proposed interventions are theoretically derived from TDF-BCT mapping and lack empirical validation. Finally, given the rapid evolution of AI technology and its regulatory context, the findings represent a snapshot in time and require ongoing re-evaluation as the field advances.

### Conclusions

This study uses a mixed methods approach to explore key factors influencing patient acceptance of health care AI. Integrating the UTAUT2 model with the theoretical domains of the TDF systematically identifies barriers and facilitators, emphasizing the need for tailored implementation strategies. Theory-based interventions are proposed by mapping the TDF to BCTs. Findings highlight that building trust in technology and prioritizing human-centered design are essential for acceptance, while privacy and ethical concerns remain significant barriers. Further research is needed to assess the effectiveness and sustainability of patient-centered, multicomponent interventions and to establish a dynamic monitoring system for evolving patient perceptions amid technological advancements.

## Supplementary material

10.2196/80581 Multimedia Appendix 1 Detailed literature search strategies, quality assessment results, and extracted raw data of barriers and facilitators.

10.2196/80581 Checklist 1 PRISMA checklist.

## Abbreviations

AI: artificial intelligence
BCT: behavior change technique
MMAT: Mixed Methods Appraisal Tool
PRISMA: Preferred Reporting Items for Systematic Reviews and Meta-Analyses
PRISMA-S: Preferred Reporting Items for Systematic Reviews and Meta-Analyses extension for Searching
TDF: Theoretical Domains Framework
UTAUT2: Unified Theory of Acceptance and Use of Technology 2

## References

[1] Aung YYM, Wong DCS, Ting DSW (2021). The promise of artificial intelligence: a review of the opportunities and challenges of artificial intelligence in healthcare. Br Med Bull.

[2] Liu R, Rong Y, Peng Z (2020). A review of medical artificial intelligence. Global Health Journal.

[3] Clark HB, Egger J, Duffy VG AI in healthcare and medicine: a systematic literature review and reappraisal.

[4] Krittanawong C (2018). The rise of artificial intelligence and the uncertain future for physicians. Eur J Intern Med.

[5] Ibrahim F, Münscher JC, Daseking M, Telle NT (2024). The technology acceptance model and adopter type analysis in the context of artificial intelligence. Front Artif Intell.

[6] Chismar WG, Wiley-Patton S Does the extended technology acceptance model apply to physicians.

[7] Liyanage H, Liaw ST, Jonnagaddala J (2019). Artificial intelligence in primary health care: perceptions, issues, and challenges. Yearb Med Inform.

[8] Kovarik CL (2020). Patient perspectives on the use of artificial intelligence. JAMA Dermatol.

[9] Ayad N, Schwendicke F, Krois J (2023). Patients’ perspectives on the use of artificial intelligence in dentistry: a regional survey. Head Face Med.

[10] Moy S, Irannejad M, Manning SJ (2024). Patient perspectives on the use of artificial intelligence in health care: a scoping review. J Patient Cent Res Rev.

[11] Alsanosi SM, Aldajani AQ, Gheliwi HA (2025). Knowledge, attitudes, and perceptions of chronic patients in Saudi Arabia regarding the use of artificial intelligence to improve medication adherence. Patient Prefer Adherence.

[12] Gundlack J, Negash S, Thiel C (2025). Artificial intelligence in medical care - patients’ perceptions on caregiving relationships and ethics: a qualitative study. Health Expect.

[13] Krisnan L, Masrom M, Yahya Y, Krishnan M (2025). Patient perceptions toward the application of artificial intelligence in diabetes care: a qualitative study. Asian J Soc Health Behav.

[14] Venkatesh V, Morris MG, Davis GB, Davis FD (2003). User acceptance of information technology: toward a unified view. MIS Q.

[15] Schomakers EM, Lidynia C, Vervier LS, Calero Valdez A, Ziefle M (2022). Applying an extended UTAUT2 model to explain user acceptance of lifestyle and therapy mobile health apps: survey study. JMIR Mhealth Uhealth.

[16] Schmitz A, Díaz-Martín AM, Yagüe Guillén MJ (2022). Modifying UTAUT2 for a cross-country comparison of telemedicine adoption. Comput Human Behav.

[17] Wang H, Tao D, Yu N, Qu X (2020). Understanding consumer acceptance of healthcare wearable devices: an integrated model of UTAUT and TTF. Int J Med Inform.

[18] Palas JU, Sorwar G, Hoque MR, Sivabalan A (2022). Factors influencing the elderly’s adoption of mHealth: an empirical study using extended UTAUT2 model. BMC Med Inform Decis Mak.

[19] Chang YT, Chao CM, Yu CW, Lin FC (2021). Extending the utility of UTAUT2 for hospital patients’ adoption of medical apps: moderating effects of e-health literacy. Mob Inf Syst.

[20] Cane J, O’Connor D, Michie S (2012). Validation of the theoretical domains framework for use in behaviour change and implementation research. Implement Sci.

[21] Cane J, Richardson M, Johnston M, Ladha R, Michie S (2015). From lists of behaviour change techniques (BCTs) to structured hierarchies: comparison of two methods of developing a hierarchy of BCTs. Br J Health Psychol.

[22] Page MJ, McKenzie JE, Bossuyt PM (2021). The PRISMA 2020 statement: an updated guideline for reporting systematic reviews. BMJ.

[23] Rethlefsen ML, Kirtley S, Waffenschmidt S (2021). PRISMA-S: an extension to the PRISMA Statement for Reporting Literature Searches in Systematic Reviews. Syst Rev.

[24] Lockwood C, Munn Z, Porritt K (2015). Qualitative research synthesis: methodological guidance for systematic reviewers utilizing meta-aggregation. Int J Evid Based Healthc.

[25] Hong QN, Fàbregues S, Bartlett G (2018). The Mixed Methods Appraisal Tool (MMAT) version 2018 for information professionals and researchers. EFI.

[26] Aromataris E, L C, Porritt K, Pilla B, Jordan Z (2024). JBI manual for evidence synthesis. JBI.

[27] Thomas J, Harden A (2008). Methods for the thematic synthesis of qualitative research in systematic reviews. BMC Med Res Methodol.

[28] Popay J, Roberts H, Sowden A (2006). Guidance on the conduct of narrative synthesis in systematic reviews: a product from the ESRC Methods Programme.

[29] Camacho J, Zanoletti-Mannello M, Landis-Lewis Z, Kane-Gill SL, Boyce RD (2020). A conceptual framework to study the implementation of clinical decision support systems (BEAR): literature review and concept mapping. J Med Internet Res.

[30] Venkatesh V, Thong JYL, Xu X (2012). Consumer acceptance and use of information technology: extending the unified theory of acceptance and use of technology. MIS Q.

[31] Di Castelnuovo A, Bonaccio M, Costanzo S (2020). Common cardiovascular risk factors and in-hospital mortality in 3,894 patients with COVID-19: survival analysis and machine learning-based findings from the multicentre Italian CORIST Study. Nutr Metab Cardiovasc Dis.

[32] (2012). Benefits and complication of peripherally inserted central catheter (PICC) versus peripherally intravenous vein lines. World Health Organization.

[33] Atkins L, Francis J, Islam R (2017). A guide to using the Theoretical Domains Framework of behaviour change to investigate implementation problems. Implement Sci.

[34] Michie S, Richardson M, Johnston M (2013). The behavior change technique taxonomy (v1) of 93 hierarchically clustered techniques: building an international consensus for the reporting of behavior change interventions. Ann Behav Med.

[35] Michie S, Atkins L, Wes R (2014). The Behaviour Change Wheel: A Guide to Developing Interventions.

[36] Huang W, Ong WC, Wong MKF (2024). Applying the UTAUT2 framework to patients’ attitudes toward healthcare task shifting with artificial intelligence. BMC Health Serv Res.

[37] Haan M, Ongena YP, Hommes S, Kwee TC, Yakar D (2019). A qualitative study to understand patient perspective on the use of artificial intelligence in radiology. J Am Coll Radiol.

[38] Jalil S, Myers T, Atkinson I, Soden M (2019). Complementing a clinical trial with human-computer interaction: patients’ user experience with telehealth. JMIR Hum Factors.

[39] Adams SJ, Tang R, Babyn P (2020). Patient perspectives and priorities regarding artificial intelligence in radiology: opportunities for patient-centered radiology. J Am Coll Radiol.

[40] Nelson CA, Pérez-Chada LM, Creadore A (2020). Patient perspectives on the use of artificial intelligence for skin cancer screening: a qualitative study. JAMA Dermatol.

[41] Benrimoh D, Tanguay-Sela M, Perlman K (2021). Using a simulation centre to evaluate preliminary acceptability and impact of an artificial intelligence-powered clinical decision support system for depression treatment on the physician-patient interaction. BJPsych Open.

[42] Musbahi O, Syed L, Le Feuvre P, Cobb J, Jones G (2021). Public patient views of artificial intelligence in healthcare: a nominal group technique study. Digit Health.

[43] Zhang Z, Citardi D, Wang D, Genc Y, Shan J, Fan X (2021). Patients’ perceptions of using artificial intelligence (AI)-based technology to comprehend radiology imaging data. Health Informatics J.

[44] Mikkelsen JG, Sørensen NL, Merrild CH, Jensen MB, Thomsen JL (2023). Patient perspectives on data sharing regarding implementing and using artificial intelligence in general practice - a qualitative study. BMC Health Serv Res.

[45] Pelayo C, Hoang J, Mora Pinzón M (2023). Perspectives of Latinx patients with diabetes on teleophthalmology, artificial intelligence-based image interpretation, and virtual care: a qualitative study. Telemed Rep.

[46] Pelly M, Fatehi F, Liew D, Verdejo-Garcia A (2023). Artificial intelligence for secondary prevention of myocardial infarction: a qualitative study of patient and health professional perspectives. Int J Med Inform.

[47] Robertson C, Woods A, Bergstrand K, Findley J, Balser C, Slepian MJ (2023). Diverse patients’ attitudes towards artificial intelligence (AI) in diagnosis. PLOS Digit Health.

[48] Neves MVM, Furlan L, Fregni F (2023). Robotic-assisted gait training (RAGT) in stroke rehabilitation: a pilot study. Arch Rehabil Res Clin Transl.

[49] Godoy Junior CA, Miele F, Mäkitie L (2024). Attitudes toward the adoption of remote patient monitoring and artificial intelligence in Parkinson’s disease management: perspectives of patients and neurologists. Patient.

[50] Maris MT, Koçar A, Willems DL (2024). Ethical use of artificial intelligence to prevent sudden cardiac death: an interview study of patient perspectives. BMC Med Ethics.

[51] Sachdeva M, Datchoua AM, Yakam VF (2024). Acceptability of artificial intelligence for cervical cancer screening in Dschang, Cameroon: a qualitative study on patient perspectives. Reprod Health.

[52] Tursynbek A, Zhaksylykova D, Cruz JP, Balay-Odao EM (2024). Perspectives of patients regarding artificial intelligence and its application in healthcare: a qualitative study. J Clin Nurs.

[53] Gundlack J, Thiel C, Negash S (2025). Patients’ perceptions of artificial intelligence acceptance, challenges, and use in medical care: qualitative study. J Med Internet Res.

[54] Trivedi R, Shaw T, Sheahen B, Chow CK, Laranjo L (2025). Patient perspectives on conversational artificial intelligence for atrial fibrillation self-management: qualitative analysis. J Med Internet Res.

[55] Sebastian G, George A, Jackson G (2023). Persuading patients using rhetoric to improve artificial intelligence adoption: experimental study. J Med Internet Res.

[56] Zhou Y, Shi Y, Lu W, Wan F (2022). Did artificial intelligence invade humans? The study on the mechanism of patients’ willingness to accept artificial intelligence medical care: from the perspective of intergroup threat theory. Front Psychol.

[57] Yang K, Zeng Z, Peng H, Jiang Y (2019). Attitudes of Chinese cancer patients toward the clinical use of artificial intelligence. Patient Prefer Adherence.

[58] Jutzi TB, Krieghoff-Henning EI, Holland-Letz T (2020). Artificial intelligence in skin cancer diagnostics: the patients’ perspective. Front Med (Lausanne).

[59] Meyer AND, Giardina TD, Spitzmueller C, Shahid U, Scott TMT, Singh H (2020). Patient perspectives on the usefulness of an artificial intelligence-assisted symptom checker: cross-sectional survey study. J Med Internet Res.

[60] Ongena YP, Haan M, Yakar D, Kwee TC (2020). Patients’ views on the implementation of artificial intelligence in radiology: development and validation of a standardized questionnaire. Eur Radiol.

[61] Aggarwal R, Farag S, Martin G, Ashrafian H, Darzi A (2021). Patient perceptions on data sharing and applying artificial intelligence to health care data: cross-sectional survey. J Med Internet Res.

[62] Esmaeilzadeh P, Mirzaei T, Dharanikota S (2021). Patients’ perceptions toward human-artificial intelligence interaction in health care: experimental study. J Med Internet Res.

[63] Liu T, Tsang W, Huang F (2021). Patients’ preferences for artificial intelligence applications versus clinicians in disease diagnosis during the SARS-CoV-2 pandemic in China: discrete choice experiment. J Med Internet Res.

[64] Lennartz S, Dratsch T, Zopfs D (2021). Use and control of artificial intelligence in patients across the medical workflow: single-center questionnaire study of patient perspectives. J Med Internet Res.

[65] Armero W, Gray KJ, Fields KG, Cole NM, Bates DW, Kovacheva VP (2022). A survey of pregnant patients’ perspectives on the implementation of artificial intelligence in clinical care. J Am Med Inform Assoc.

[66] Yulan Z, Shu X, Yang Z, Chen Q (2022). An exploration of the current status and influencing factors of elderly patients’ willingness to use artificial intelligence robots. West China Medical Journal.

[67] Khullar D, Casalino LP, Qian Y, Lu Y, Krumholz HM, Aneja S (2022). Perspectives of patients about artificial intelligence in health care. JAMA Netw Open.

[68] Kosan E, Krois J, Wingenfeld K, Deuter CE, Gaudin R, Schwendicke F (2022). Patients’ perspectives on artificial intelligence in dentistry: a controlled study. J Clin Med.

[69] Kawsar A, Hussain K, Kalsi D, Kemos P, Marsden H, Thomas L (2023). Patient perspectives of artificial intelligence as a medical device in a skin cancer pathway. Front Med (Lausanne).

[70] Mahlknecht A, Engl A, Piccoliori G, Wiedermann CJ (2023). Supporting primary care through symptom checking artificial intelligence: a study of patient and physician attitudes in Italian general practice. BMC Prim Care.

[71] Parry MW, Markowitz JS, Nordberg CM, Patel A, Bronson WH, DelSole EM (2023). Patient perspectives on artificial intelligence in healthcare decision making: a multi-center comparative study. Indian J Orthop.

[72] Temple S, Rowbottom C, Simpson J (2023). Patient views on the implementation of artificial intelligence in radiotherapy. Radiography (Lond).

[73] Baghdadi LR, Mobeirek AA, Alhudaithi DR (2024). Patients’ attitudes toward the use of artificial intelligence as a diagnostic tool in radiology in Saudi Arabia: cross-sectional study. JMIR Hum Factors.

[74] Bahadir HS, Keskin NB, Çakmak EŞK, Güneç G, Cesur Aydin K, Peker F (2025). Patients’ attitudes toward artificial intelligence in dentistry and their trust in dentists. Oral Radiol.

[75] Esin H, Karaali C, Teker K (2024). Patients’ perspectives on the use of artificial intelligence and robots in healthcare. Bratisl Lek Listy.

[76] Fransen SJ, Kwee TC, Rouw D (2025). Patient perspectives on the use of artificial intelligence in prostate cancer diagnosis on MRI. Eur Radiol.

[77] Riedl R, Hogeterp SA, Reuter M (2024). Do patients prefer a human doctor, artificial intelligence, or a blend, and is this preference dependent on medical discipline? Empirical evidence and implications for medical practice. Front Psychol.

[78] Chan J, Parker L, Carter S, Nickel B, Carroll S (2025). Radiation oncology patients’ perceptions of artificial intelligence and machine learning in cancer care: a multi-centre cross-sectional study. Radiother Oncol.

[79] Ozcan BB, Dogan BE, Xi Y, Knippa EE (2025). Patient perception of artificial intelligence use in interpretation of screening mammograms: a survey study. Radiol Imaging Cancer.

[80] Tirapelli C, Gaêta-Araujo H, Costa ED (2025). Patient perceptions of artificial intelligence in dental imaging diagnostics: a multicentre survey. Dentomaxillofac Radiol.

[81] Xiangde L (2019). Investigation on patients’ cognition and trust in artificial intelligence medicine. Chinese Medical Ethics.

[82] Menghan W, Weihua F (2020). Influence factors of public willingness to use internet medical service platform. J Beijing Univ Aero & Astro (Social Sciences Edition).

[83] Siwen Z, Jiudi Z (2022). Investigation on the acceptance of health education robots among patients, family members, and medical staff in esophageal cancer surgery. Modern Nurse · Special Edition.

[84] Jin FS (2023). Development and acceptance of a virtual reality system for rehabilitation training of swallowing disorders in stroke patients. Chinese Journal of Modern Nursing.

[85] Haggenmüller S, Maron RC, Hekler A (2024). Patients’ and dermatologists’ preferences in artificial intelligence-driven skin cancer diagnostics: a prospective multicentric survey study. J Am Acad Dermatol.

[86] Macri CZ, Bacchi S, Wong W, Baranage D, Sivagurunathan PD, Chan WO (2024). A pilot survey of patient perspectives on an artificial intelligence-generated presenter in a patient information video about face-down positioning after vitreoretinal surgery. Ophthalmic Res.

[87] Palmisciano P, Jamjoom AAB, Taylor D, Stoyanov D, Marcus HJ (2020). Attitudes of patients and their relatives toward artificial intelligence in neurosurgery. World Neurosurg.

[88] van der Zander QEW, van der Ende-van Loon MCM, Janssen JMM (2022). Artificial intelligence in (gastrointestinal) healthcare: patients’ and physicians’ perspectives. Sci Rep.

[89] Katirai A, Yamamoto BA, Kogetsu A, Kato K (2023). Perspectives on artificial intelligence in healthcare from a Patient and Public Involvement Panel in Japan: an exploratory study. Front Digit Health.

[90] Gonzalez XT, Steger-May K, Abraham J (2025). Just another tool in their repertoire: uncovering insights into public and patient perspectives on clinicians’ use of machine learning in perioperative care. J Am Med Inform Assoc.

[91] Witkowski K, Dougherty RB, Neely SR (2024). Public perceptions of artificial intelligence in healthcare: ethical concerns and opportunities for patient-centered care. BMC Med Ethics.

[92] Limayem M, Hirt SG, Cheung CMK (2007). How habit limits the predictive power of intention: the case of information systems continuance. MIS Q.

[93] Brown SA, Venkatesh V (2005). Model of adoption of technology in households: a baseline model test and extension incorporating household life cycle. MIS Q.

[94] Robinson R, Liday C, Lee S (2023). Artificial intelligence in health care-understanding patient information needs and designing comprehensible transparency: qualitative study. JMIR AI.

[95] Change Z, Huiling Z, Huiyin Z (2019). Study on the influence mechanism of the use of hospital intelligent medical system on patients’ satisfaction: based on the perspective of technology acceptance model. Chinese Hospital Management.

[96] Kai W (2023). Robot-assisted gait training experience in stroke patients: A qualitative research. Military Nursing.

[97] Chengfu Z, Xiangzhou W Risk challenges of embedding artificial intelligence in public service governance. E-Government.

[98] Almokdad E, Mouloudj K, Lee CH (2025). Rehumanizing AI-driven service: how employee presence shapes consumer perceptions in digital hospitality settings. JTAER.

[99] Xiong JF, Jia TY, Li XY (2018). Identifying epidermal growth factor receptor mutation status in patients with lung adenocarcinoma by three-dimensional convolutional neural networks. Br J Radiol.

