# Supplementary material for: Barriers and Facilitators to Patient Acceptance of Artificial Intelligence in Health Care: Systematic Review
Source: J Med Internet Res. 2026 May 8;28:e80581. doi: 10.2196/80581 (PMC13154726; doi:10.2196/80581)
Supplement: Multimedia Appendix 1 [file jmir-v28-e80581-s001.docx]

**Table S1**. Search strategy.

**Strategies in English databases**

| PubMed 2025.12.23 | | Results |
| --- | --- | --- |
| #1 | (Artificial Intelligence[MeSH Terms] OR (Machine Learning[Title/Abstract] OR Deep Learning[Title/Abstract] OR Neural Network[Title/Abstract] OR Technol* System[Title/Abstract] OR Smart System[Title/Abstract] OR Intelligent System[Title/Abstract] OR Assistive System[Title/Abstract] OR Decision Support System[Title/Abstract] OR Human–Computer Interaction[Title/Abstract] OR Human Machine Interaction[Title/Abstract] OR Cognitive System[Title/Abstract] OR Decision Engineering[Title/Abstract] OR Natural Language Understanding[Title/Abstract]) | 450,542 |
| #2 | Patients[MeSH Terms] | 90,217 |
| #3 | (attitude OR behaviour OR perception)[MeSH Terms] OR view*[Title/Abstract] OR perspective*[Title/Abstract] OR experience*[Title/Abstract] OR perception*[Title/Abstract] OR barrier*[Title/Abstract] OR challeng*[Title/Abstract] OR facilitator*[Title/Abstract] OR understand*[Title/Abstract] OR enabler*[Title/Abstract] OR belief*[Title/Abstract] OR attitude*[Title/Abstract] OR behavior*[Title/Abstract] OR enabler*[Title/Abstract] OR motivator*[Title/Abstract] OR implementation[Title/Abstract] OR compliance[Title/Abstract] OR support[Title/Abstract] OR factor*[Title/Abstract] OR attendance[Title/Abstract] OR predictor*[Title/Abstract] OR preference*[Title/Abstract]) | 14,674,937 |
| #4 | #1 AND #2 AND #3 | 475 |
| Web of Science 2025.12.23 | |  |
| #1 | TI: "Artificial Intelligence" OR "Machine Learning" OR "Deep Learning" OR "Neural Network" OR "Technol* System" OR "Smart System" OR "Intelligent System" OR "Assistive System" OR "Decision Support System" OR "Human–Computer Interaction" OR "Human Machine Interaction" OR "Cognitive System" OR "Decision Engineering" OR "Natural Language Understanding" | 780,348 |
| #2 | TI: Patients | [3,380,218](https://webvpn.zju.edu.cn/https/77726476706e69737468656265737421e7f2439321236b597b068aa9d6562f34899051d9fc85a85327/wos/woscc/summary/e52994c1-e196-41b7-85ab-54fe9d695792-0193059d3e/relevance/1) |
| #3 | TI: "Attitude" OR "Behavior" OR "Perception" OR "view*" OR "perspective*" OR "experience*" OR "perception*" OR "barrier*" OR "challeng*" OR "facilitator*" OR "understand*" OR "enabler*" OR "belief*" OR "attitude*" OR "behavior*" OR "motivator*" OR "implementation" OR "compliance" OR "support" OR "factor*" OR "attendance" OR "predictor*" OR "preference*" | 5,746,252 |
| #4 | #1 AND #2 AND #3 | 1,922 |
| EBSCO CINAHL Plus 2025.12.23 | |  |
| S1 | MH ("Artificial Intelligence" ) OR AB ( Machine Learning OR Deep Learning OR Neural Network OR Technol* System OR Smart System OR Intelligent System OR Assistive System OR Decision Support System OR Human–Computer Interaction OR Human Machine Interaction OR Cognitive System OR Decision Engineering OR Natural Language Understanding) | 48,484 |
| S2 | MH (Patients) | 11,132 |
| S3 | MH (attitude OR behaviour OR perception）OR AB (view* OR perspective* OR experience* OR perception* OR barrier* OR challeng* OR facilitator* OR understand* OR enabler* OR belief* OR attitude* OR behavior* OR enabler* OR motivator* OR implementation OR compliance OR support OR factor* OR attendance OR predictor* OR preference* ) | 2,685,307 |
| S4 | S1 AND S2 AND S3 | 28 |
| EMBASE 2025.12.23 | |  |
| #1 | “Artificial intelligence”'/exp OR (Machine Learning OR Deep Learning OR Neural Network OR Technol* System OR Smart System OR Intelligent System OR Assistive System OR Decision Support System OR Human Computer Interaction OR Human Machine Interaction OR Cognitive System OR Decision Engineering OR Natural Language Understanding).ti,ab,kw | 363,124 |
| #2 | 'patients'/exp | 3,221,291 |
| #3 | ('attitude'/exp OR 'behaviour'/exp OR 'perception'/exp) OR (view* or perspective* or experience* or perception* or barrier* or challeng* or facilitator* or understand* or enabler* or belief* or attitude* or behavior* or enabler* or motivator* or implementation or compliance or support or factor* or attendance or predictor* or preference*):ti,ab,kw | 16,900,130 |
| #4 | #1 AND #2 AND #3 | 2992 |
| Cochrane Library 2025.12.23 | |  |
| #1 | MeSH descriptor: [Artificial Intelligence] explode all trees | 3,835 |
| #2  #3 | (Machine Learning OR Deep Learning OR Neural Network OR Technol* System OR Smart System OR Intelligent System OR Assistive System OR Decision Support System OR Human–Computer Interaction OR Human Machine Interaction OR Cognitive System OR Decision Engineering OR Natural Language Understanding):ti,ab,kw  #1 OR #2 | 34,108  1,770 |
| #4 | MeSH descriptor: [Patients] explode all trees | 4,586 |
| #5 | MeSH descriptor: [Attitude] explode all trees | 55,623 |
| #6  #7  #8  #9 | MeSH descriptor: [behaviour] explode all trees  MeSH descriptor: [perception] explode all trees  (view* OR perspective* OR experience* OR perception* OR barrier* OR challeng* OR facilitator* OR understand* OR enabler* OR belief* OR attitude* OR behavior* OR enabler* OR motivator* OR implementation OR compliance OR support OR factor* OR attendance OR predictor* OR preference*):ti,ab,kw  #5 OR #6 OR #7 OR #8 | 24,747  24362  860,760  877,162 |
| #10 | #3 AND #4 AND #9 | 99 |

**Strategies in Chinese databases**

| CNKI 2025.12.23 | | Results |
| --- | --- | --- |
| #1 | 篇关摘（人工智能 + AI + 机器学习 + 深度学习 + 神经网络 + 技术系统 + 智能系统 + 辅助系统 + 决策支持系统 + 人机交互 + 认知系统 + CDSS） |  |
| #2 | 篇关摘（患者） |  |
| #3 | 篇关摘（接受度 + 看法 + 经历 + 反馈 + 信任度 + 意愿使用 + 观点 + 促进因素 + 障碍因素 + 感知 + 态度） |  |
| #4 | #1 AND #2 AND #3 | 668 |
| WAN FANG 2025.12.23 | | |
| #1 | 题目或关键词（人工智能 or AI or 机器学习 or 深度学习 or 神经网络 or 技术系统 or 智能系统 or 辅助系统 or 决策支持系统 or 人机交互 or 认知系统 or CDSS）模糊匹配 |  |
| #2 | 题目或关键词（患者）模糊匹配 |  |
| #3 | 题目或关键词（接受度 or 看法 or 经历 or 反馈 or 信任度 or 意愿使用 or 观点 or 促进因素 or 障碍因素 or 感知 or 态度）模糊匹配 |  |
| #4 | #1 AND #2 AND #3 | 781 |
| VIP 2025.12.23 | | |
| #1 | 题目或关键词：(人工智能 + AI + 机器学习 + 深度学习 + 神经网络 + 技术系统 + 智能系统 + 辅助系统 + 决策支持系统 + 人机交互 + 认知系统 + CDSS) |  |
| #2 | 题目或关键词：（患者） |  |
| #3 | 题目或关键词：（接受度 + 看法 + 经历 + 反馈 + 信任度 + 意愿使用 + 观点 + 促进因素 + 障碍因素 + 感知 + 态度） |  |
| #4 | #1 AND #2 AND #3 | 58 |
| SinoMed 2025.12.23 | | |
| #1 | 常用字段：（人工智能 or AI or 机器学习 or 深度学习 or 神经网络 or 技术系统 or 智能系统 or 辅助系统 or 决策支持系统 or 人机交互 or 认知系统 or CDSS) | 286899 |
| #2 | 常用字段：患者 | 5178550 |
| #3 | 常用字段：接受度 or 看法 or 经历 or 反馈 or 信任度 or 意愿使用 or 观点 or 促进因素 or 障碍因素 or 感知 or 态度 | 999363 |
| #4 | #1 AND #2 AND #3 | 429 |

**Table S2.** The 2012 version of the Theoretical Domains Framework (TDF) includes the following 14 theoretical domains.

|  | **Domain** | **Definition of the Domain** | **Construct** | **Definition of the Construct** |
| --- | --- | --- | --- | --- |
| D1 | Knowledge | An awareness of the existence of something | Knowledge (including knowledge of condition/scientific rationale) | An awareness of the existence of something. |
|  |  |  | Procedural knowledge | Knowing how to do something. |
|  |  |  | Knowledge of task environment | Knowledge of the social and material context in which a task is undertaken. |
| D2 | Skills | An ability or proficiency acquired through practice | Skills | An ability or proficiency acquired through training and/or practice. |
|  |  |  | Skills development | The gradual acquisition or advancement through progressive stages of an ability or proficiency acquired through training and practice. |
|  |  |  | Competence | One's repertoire of skills, and ability especially as it is applied to a task or set of tasks. |
|  |  |  | Ability | Competence or capacity to perform a physical or mental act. Ability may be either unlearned or acquired by education and practice. |
|  |  |  | Interpersonal skills | An aptitude enabling a person to carry on effective relationships with others, such as an ability to cooperate, to assume appropriate social responsibilities or to exhibit adequate flexibility. |
|  |  |  | Practice | Repetition of an act, behavior, or series of activities, often to improve performance or acquire a skill |
|  |  |  | Skills assessment | A judgment of the quality, worth, importance, level, or value of an ability or proficiency acquired through training and practice. |
| D3 | Social/professional role and identity | A coherent set of behaviors and displayed personal qualities of an individual in a social or work setting | Professional identity | The characteristics by which an individual is recognised relating to, connected with or befitting a particular profession. |
|  |  |  | Professional role | The behavior considered appropriate for a particular kind of work or social position. |
|  |  |  | Social identity | The set of behavioral or personal characteristics by which an individual is recognizable [and portrays] as a member of a social group. |
|  |  |  | Identity | An individual's sense of self defined by a) a set of physical and psychological characteristics that is not wholly shared with any other person and b) a range of social and interpersonal affiliations (e.g., ethnicity) and social roles. |
|  |  |  | Professional boundaries | The bounds or limits relating to, or connected with a particular profession or calling. |
|  |  |  | Professional confidence | An individual's belief in his or her repertoire of skills, and ability especially as it is applied to a task or set of tasks. |
|  |  |  | Group identity | The set of behavioral or personal characteristics by which an individual is recognizable [and portrays] as a member of a group. |
|  |  |  | Leadership | The processes involved in leading others, including organizing, directing, coordinating and motivating their efforts toward achievement of certain group or organization goals. |
|  |  |  | Organizational commitment | An employee's dedication to an organization and wish to remain part of it. Organizational commitment is often described as having both an emotional or moral element and a more prudent element. |
| D4 | Beliefs about capabilities | Acceptance of the truth, reality, or validity about an ability, talent, or facility that a person can put to constructive use | Self-confidence | Self-assurance or trust in one's own abilities, capabilities and judgment. |
|  |  |  | Perceived competence | An individual's belief in his or her ability to learn and execute skills. |
|  |  |  | Self-efficacy | An individual's capacity to act effectively to bring about desired results, as perceived by the individual. |
|  |  |  | Perceived behavioral control | An individual's perception of the ease or difficulty of performing the behavior of interest. |
|  |  |  | Beliefs | The thing believed; the proposition or set of propositions held true. |
|  |  |  | Self-esteem | The degree to which the qualities and characteristics contained in one's self- concept are perceived to be positive. |
|  |  |  | Empowerment | The promotion of the skills, knowledge and confidence necessary to take great control of one's life as in certain educational or social schemes; the delegation of increased decision-making powers to individuals or groups in a society or organization. |
|  |  |  | Professional confidence | An individual's belief in his or her repertoire of skills, and ability especially as it is applied to a task or set of tasks. |
| D5 | Optimism | The confidence that things will happen for the best or that desired goals will be attained | Optimism | The attitude that outcomes will be positive and that people's wishes or aims will ultimately be fulfilled. |
|  |  |  | Pessimism | The attitude that things will go wrong and that people's wishes or aims are unlikely to be fulfilled. |
|  |  |  | Unrealistic optimism | The inert tendency for humans to over-rate their own abilities and chances of positive outcomes compared to those of other people. |
|  |  |  | Identity | An individual's sense of self defined by a) a set of physical and psychological characteristics that is not wholly shared with any other person and b) a range of social and interpersonal affiliations (e.g., ethnicity) and social roles. |
| D6 | Belief about consequences | Acceptance of the truth, reality, or validity about outcomes of a behavior in a given situation | Beliefs | The thing believed; the proposition or set of propositions held true. |
|  |  |  | Outcome expectancy | behaviors |
|  |  |  | Characteristics of outcome expectancy | Characteristics of the cognitive, emotional and behavioral outcomes that individuals believe are associated with future or intended behaviors and that are believed to either promote or inhibit these behaviors These include whether they are sanctions/rewards, proximal/distal, valued/not valued, probable/improbable, salient/not salient, perceived risks or threats. |
|  |  |  | Anticipated regret | A sense of the potential negative consequences of a decision that influences the choice made: for example an individual may decide not to make an investment because of the feelings associated with an imagined loss. |
|  |  |  | Consequent | An outcome of behavior in a given situation. |
| D7 | Reinforcement | Increasing the probability of a response by arranging a dependent relationship, or contingency, between the response and a given stimulus | Rewards (proximal / distal, valued / not valued, probable / improbable) | Return or recompense made to, or received by a person contingent on some performance. |
|  |  |  | Incentives | An external stimulus, such as condition or object, that enhances or serves as a motive for behavior. |
|  |  |  | Punishment | The process in which the relationship between a response and some stimulus or circumstance results in the response becoming less probable; a painful, unwanted or undesired event or circumstance imposed as a penalty on a wrongdoer. |
|  |  |  | Consequent | An outcome of behavior in a given situation. |
|  |  |  | Reinforcement | A process in which the frequency of a response is increased by a dependent relationship or contingency with a stimulus. |
|  |  |  | Contingencies | A conditional probabilistic relation between two events. Contingencies may be arranged via dependencies or they may emerge by accident. |
|  |  |  | Sanctions | A punishment or other coercive measure, usually administered by a recognized authority, that is used to penalize and deter inappropriate or unauthorized actions. |
| D8 | Intentions | A conscious decision to perform a behavior or a resolve to act in a certain way | Stability of intentions | Ability of one's resolve to remain in spite of disturbing influences. |
|  |  |  | Stages of change model | A model that proposes that behavior change is accomplished through five specific stages: Pr-contemplation, Contemplation, Preparation, Action, and Maintenance. |
|  |  |  | Trans-theoretical model and stages of change | A five-stage theory to explain changes in people's health behavior. It suggests that change takes time, that different interventions are effective at different stages, and that there are multiple outcomes occurring across the stages. |
| D9 | Goals | Mental representation of outcomes or end states that an individual wants to achieve | Goals (distal / proximal) | Desired state of affairs of a person or system, these may be closer (proximal) or further away (distal). |
|  |  |  | Goal priority | Order of importance or urgency of end states toward which one is striving. |
|  |  |  | Goal / target setting | A process that establishes specific time based behavior targets that are measurable, achievable and realistic. |
|  |  |  | Goals (autonomous / controlled) | The end state toward which one is striving: the purpose of an activity or endeavor. It can be identified by observing that a person ceases or changes its behavior upon attaining this state; proficiency in a task to be achieved within a set period of time. |
|  |  |  | Action planning | The action or process of forming a plan regarding a thing to be done or a deed. |
|  |  |  | Implementation intention | The plan that one creates in advance of when, where and how one will enact a behavior. |
| D10 | Memory, attention, and decision processes | The ability to retain information, focus selectively on aspects of the environment and choose between two or more alternatives | Memory | The ability to retain information or a representation of a past experience, based on the mental processes of learning or encoding retention across some interval of time, and retrieval or reactivation of the memory; specific information of a specific past. |
|  |  |  | Attention | A state of awareness in which the senses are focused selectively on aspects of the environment and the central nervous system is in a state of readiness to respond to stimuli. |
|  |  |  | Attention control | The extent to which a person can concentrate on relevant cues and ignore all irrelevant cues in a given situation. |
|  |  |  | Decision making | The cognitive process of choosing between two or more alternatives, ranging from the relatively clear cut to the complex. |
|  |  |  | Cognitive overload / tiredness | The situation in which the demands placed on a person by mental work are greater than a person's mental abilities. |
| D11 | Environmental context and resources | Any circumstances of a person’s situation or environment that discourages or encourages the development of skills and abilities, independence, social competence, and adaptive behavior | Environmental stressors | External factors in the environment that cause stress. |
|  |  |  | Resources / material resources | Commodities and human resources used in enacting a behavior. |
|  |  |  | Organizational culture /climate | A distinctive pattern of thought and behavior shared by members of the same organization and reflected in their language, values, attitudes, beliefs, and customs. |
|  |  |  | Salient events / critical incidents | Occurrences that one judges to be distinctive, prominent or otherwise significant. |
|  |  |  | Person x environment interaction | Interplay between the individual and their surroundings. |
|  |  |  | Barriers and facilitators | In psychological contexts barriers/facilitators are mental, emotional or behavioral limitations/strengths in individuals or groups. |
| D12 | Social influences | Those interpersonal processes that can cause individuals to change their thoughts, feelings, or behaviors | Social pressure | The exertion of influence on a person or group by another person or group. |
|  |  |  | Social norms | Socially determined consensual standards that indicate a) what behaviors are considered typical in a given context and b) what behaviors are considered proper in the context. |
|  |  |  | Group conformity | The act of consciously maintaining a certain degree of similarity to those in your general social circles. |
|  |  |  | Social comparisons | The process by which people evaluate their attitudes, abilities, or performance relative to others. |
|  |  |  | Group norms | Any behavior, belief, attitude or emotional reaction held to be correct or acceptable by a given group in society. |
|  |  |  | Social support | The apperception or provision of assistance or comfort to others, typically in order to help them cope with a variety of biological, psychological and social stressors. Support may arise from any interpersonal relationship in an individual's social network, involving friends, neighbors, religious institutions, colleagues, caregivers or support groups. |
|  |  |  | Power | The capacity to influence others, even when they try to resist this influence. |
|  |  |  | Inter group conflict | Disagreement or confrontation between two or more groups and their members. This may involve physical violence, interpersonal discord, or psychological tension. |
|  |  |  | Alienation | Estrangement from one's social group; a deep seated sense of dissatisfaction with one's personal experiences that can be a source of lack of trust in one's social or physical environment or in oneself; the experience of separation between thoughts and feelings. |
|  |  |  | Group identity | The set of behavioral or personal characteristics by which an individual is recognizable [and portrays] as a member of a group. |
|  |  |  | Modeling | In developmental psychology the process in which one or more individuals or other entities serve as examples (models) that a child will copy. |
| D13 | Emotion | A complex reaction pattern, involving experiential, behavioral, and physiological elements, by which the individual attempts to deal with a personally significant matter or event | Fear | An intense emotion aroused by the detection of imminent threat, involving an immediate alarm reaction that mobilizes the organism by triggering a set of physiological changes. |
|  |  |  | Anxiety | A mood state characterized by apprehension and somatic symptoms of tension in which an individual anticipates impending danger, catastrophe or misfortune. |
|  |  |  | Affect | An experience or feeling of emotion, ranging from suffering to elation, from the simplest to the most complex sensations of feelings, and from the most normal to the most pathological emotional reactions. |
|  |  |  | Stress | A state of physiological or psychological response to internal or external stressors. |
|  |  |  | Depression | A mental state that presents with depressed mood, loss of interest or pleasure, feelings of guilt or low self-worth, disturbed sleep or appetite, low energy, and poor concentration. |
|  |  |  | Positive / negative affect | The internal feeling/state that occurs when a goal has/has not been attained, a source of threat has/has not been avoided, or the individual is/is not satisfied with the present state of affairs. |
|  |  |  | Burn-out | Physical, emotional or mental exhaustion, especially in one's job or career, accompanied by decreased motivation, lowered performance and negative attitudes towards oneself and others. |
| D14 | Behavioral regulation | Anything aimed at managing or changing objectively observed or measured actions | Self-monitoring | A method used in behavioral management in which individuals keep a record of their behavior especially in connection with efforts to change or regulate the self; a personality trait reflecting an ability to modify one's behaviorism response to situation. |
|  |  |  | Breaking habit | To discontinue a behavior or sequence of behaviorist is automatically activated by relevant situational cues. |
|  |  |  | Action planning | The action or process of forming a plan regarding a thing to be done or a deed. |

**Table S3.** BCT Taxonomy (v1): 93 hierarchically clustered techniques.

| Grouping and BCTs | Grouping and BCTs | Grouping and BCTs |
| --- | --- | --- |
| 1. Goals and planning  Goal setting (behavior)  Problem solving  Goal setting (outcome)  Action planning  Review behavior goal(s)  Discrepancy between current behavior and goal  Review outcome goal(s)  Behavioral contract  Commitment  2. Feedback and monitoring  2.1. Monitoring of behavior by others without feedback  2.2. Feedback on behavior  2.3. Self-monitoring of behavior  2.4. Self-monitoring of outcome(s) of behavior  2.5. Monitoring of outcome(s) of behavior without feedback  2.6. Biofeedback  2.7. Feedback on outcome(s) of behavior  3. Social support  3.1. Social support (unspecified)  3.2. Social support (practical)  3.3. Social support (emotional)  4. Shaping knowledge  4.1. Instruction on how to perform the behavior  4.2. Information about Antecedents  4.3. Re-attribution  4.4. Behavioral experiments  5. Natural consequences  5.1. Information about health  consequences  5.2. Salience of consequences  5.3. Information about social and environmental consequences  5.4. Monitoring of emotional consequences  5.5. Anticipated regret  5.6. Information about emotional consequences | 6. Comparison of behavior  6.1. Demonstration of the behavior  6.2. Social comparison  6.3. Information about others’ approval  7. Associations  7.1. Prompts/cues  7.2. Cue signaling reward  7.3. Reduce prompts/cues  7.4. Remove access to the reward  7.5. Remove aversive stimulus  7.6. Satiation  7.7. Exposure  7.8. Associative learning  8. Repetition and substitution  8.1. Behavioral practice/rehearsal  8.2. Behavior substitution  8.3. Habit formation  8.4. Habit reversal  8.5. Over-correction  8.6. Generations of target behavior  8.7. Graded tasks  9. Comparison of outcomes  9.1. Credible source  9.2. Pros and cons  9.3. Comparative imagining of future outcomes  10. Reward and threat  10.1. Material incentive (behavior)  10.2. Material reward (behavior)  10.3. Non-specific reward  10.4. Social reward  10.5. Social incentive  10.6. Non-specific incentive  10.7. Self-incentive  10.8. Incentive (outcome)  10.9. Self-reward  10.10. Reward (outcome)  10.11. Future punishment  11. Regulation  11.1. Pharmacological support  11.2. Reduce negative emotions  11.3. Conserving mental resources  11.4. Paradoxical instructions | 12. Antecedents  12.1. Restructuring the physical environment  12.2. Restructuring the social environment  12.3. Avoidance/reducing exposure to cues for the behavior  12.4. Distraction  12.5. Adding objects to the environment  12.6. Body changes  13. Identity  13.1. Identification of self as role model  13.2. Framing/re framing  13.3. Incompatible beliefs  13.4. Valued self-identify  13.5. Identity associated with changed behavior  14. Scheduled consequences  14.1. Behavior cost  14.2. Punishment  14.3. Remove reward  14.4. Reward approximation  14.5. Rewarding completion  14.6. Situation-specific reward  14.7. Reward incompatible behavior  14.8. Reward alternative behavior  14.9. Reduce reward frequency  14.10. Remove punishment  15. Self-belief  15.1. Verbal persuasion about capability  15.2. Mental rehearsal of successful performance  15.3. Focus on past success  15.4. Self-talk  16. Covert learning  16.1. Imaginary punishment  16.2. Imaginary reward  16.3. Vicarious consequences |

**Table S4.** Operational definitions and scoring examples of the APEASE criteria in this study.

| **Dimension** | **Specific Focus in This Study (Promoting Medical AI Adoption)** | **0** | **1** | **2** |
| --- | --- | --- | --- | --- |
| Affordability | The financial, time, and human resource costs of implementing the BCT for a healthcare institution. |  |  |  |
| Practicality | Whether the BCT can be integrated into the busy existing workflows of healthcare professionals. |  |  |  |
| Effectiveness | Whether evidence suggests the BCT can improve patients' acceptance or intention to use AI. |  |  |  |
| Acceptability | Whether the BCT is perceived as appropriate, ethical, and satisfactory by patients, staff, and managers. |  |  |  |
| Side-effects | Whether implementation risks unintended negative consequences (e.g., anxiety, distrust). |  |  |  |
| Equity | Whether the benefits of the BCT are likely to reach patients with different digital literacy or socioeconomic status. |  |  |  |

**Note：**We employed a three-point scoring system **(2 = favorable/meets criteria, 1 = neutral/uncertain, 0 = unfavorable/does not meet criteria)** rather than a more granular Likert scale. This decision was based on the following considerations: First, the primary objective of this assessment stage was the feasibility categorization of BCTs, not their precise ranking. The three-point system most directly facilitates the subsequent classification into high, medium, and low feasibility. Second, given that judgments were made based on existing literature and expert consensus, the three-point scale minimizes subjective interpretation differences among raters, thereby enhancing inter-rater reliability and assessment efficiency. Finally, this simplified approach necessitates a directional judgment, which helps clearly identify strategies requiring further verification (predominantly scoring '1') or those with definite implementation barriers (scoring '0'), thus making the final output clearer and more actionable for practitioners and decision-makers.

**Table 5.** Decision rules and examples for BCT feasibility classification.

| Feasibility Class | Core Decision Rules (All Must Be Met) |
| --- | --- |
| High Feasibility | 1. No critical barriers in core implementation dimensions: No low scores (0) in Affordability (A), Practicality (P), and Acceptability (A). 2. No major risks: No critical flaws (score of 0) in Side-effects (S) and Equity (E). |
| Medium Feasibility | 1. Evidence of effectiveness: At least a medium score (≥1) in Effectiveness (E). 2. Contains manageable limitations: Challenges (score of 1 or an isolated 0) in 1-2 core implementation dimensions (A, P, A) that can be addressed with planning; OR, has minor, manageable S/E concerns. |
| Low Feasibility | Meets either of the following: 1. Major barrier in core implementation: An insurmountable barrier (score of 0) in any core implementation dimension (A, P, A). 2. Unacceptable risk: A severe and unmitigable issue (score of 0) in Side-effects (S) or Equity (E). |

**Table S6.** Features of the study and participants were included, stratified by study design.

| Author(s), year | Application forms of AI | Data collection method | Conclusion |
| --- | --- | --- | --- |
| Wang et al., 2023 | AI robot | Semi-structured interview | • Facilitate:  ①Recognition of the effectiveness of rehabilitation assistance.  ②Positive experiences with rehabilitation assistance.  ③Positive feedback that promotes an increase in self-accomplishment. • Barriers:  ①Fear of experiencing new things.  ②Frustration due to training not meeting expectations.  ③Physical discomfort during training. |
| Adams et al., 2020 | AI in Radiology | Round-table discussions | • Facilitate:  ①Reducing time to diagnosis.  ②Improving Access to Imaging and reducing wait times.  ③Increasing diagnostic accuracy.  ④Empowering patients.  ⑤Improving communication. • Barriers:  ①AI is at odds with cultural ways of knowing and healing.  ②the lack of human connection.  ③A lack of trust in AI; misconceptions about. |
| Godoy Junior et al., 2024 | AI-assisted RMS in PD care | Qualitative interviews and focus-groups | • Facilitate:  The successful uptake hinges on clear clinical advantages, user-friendliness, and maintaining trust between doctors and patients. • Barriers:  ①Privacy concerns  ②the fear of losing human touch emerged. |
| Haan et al., 2019 | AI in Radiology. | Semi-structured face-to-face inter view | • Facilitate:  ①Efficacy and reliability of AI throughout the entire process of scan evaluation.  ②Shorter amount of time.  ③Being assisted sooner and that will reduce costs. • Barriers:  Patients were rather skeptical about the skills of AI systems. |
| Jalil et al., 2019 | Telehealth | Ethnographic philosophy and semi-structured interviews | • Facilitate:  ①Feel safe  ②Reduced doctor visits. • Barriers:  ①It was difficult to use.  ②It also gave uncomfortable experiences.  ③Lack of visual data; lack of medication name.  ④Mismatch with life due to immobility of the device.  ⑤Glucometer discomfort and pain |
| Maris et al., 2024 | AI in clinical decision-making regarding the implantation of an ICD | Semi-structured, future scenario-based interviews | • Facilitate:  ①Rectify human limitations with AI.  ②Explainability, transparency and trust.  ③Serve as a second opinion. • Barriers:  ①Data cybersecurity.  ②Bias and quality of AI data.  ③Less human interaction.  ④Algorithm errors and responsibility.  ⑤Limitation in technology". |
| Mikkelsen et al., 2023 | No specific restriction | Vignette method | • Facilitate:  ①Trust in GP.  ②AI as a support tool.  ③AI's potential to improve quality of care. • Barriers:  ①Privacy concerns.  ②Fear of AI replacing human interaction.  ③AI's limitations in detecting emotional states. |
| Nelson et al., 2020 | AI in Skin Cancer Screening | Using a grounded theory approach to semi-structured interview analysis. | • Facilitate:  ①Patients valued the increased diagnostic speed (60%) and health care access (60%) provided by AI.  ②With patients envisioning AI as a tool for referring to a physician  ③Providing a second opinion for a physician. • Barriers:  ①Increased patient anxiety.  ②Concerned about the potential for false negatives, false-positives, inaccurate or limited training set,  ③Lack of context, lack of physical examination, and operator dependence. |
| Pelayo et al., 2023 | ophthalmology with AI (AI)-based image interpretation. | Semi-structured, individual interviews | • Facilitate:  ①AI as a decision support tool.  ②AI's speed and accuracy. • Barriers:  ①Distrust of new technologies.  ②Uncertain regarding the accuracy of AI.  ③Preference for human oversight.  ④Preference for in-person care.  ⑤Concerns about non-verbal communication. |
| Robinson et al., 2023 | AI/ML device and application | We conducted a series of 9 focus groups and 3 semi-structured interviews. | • Facilitate:  ①Having clear predictive and real-time alerts.  ②Trusting in the healthcare provider’s recommendations  ③The trust in the device was directly related to patient experience, device accuracy, and duration of device use; transparency, explainability, and accuracy metrics are important. • Barriers:  ①Little is known about how user interaction with specific AI/ML applications or related system information.  ②Users cannot see or interpret the alert. |
| Sachdeva et al., 2024 | automated smartphone-based image classifier | Semi-structured interview | • Facilitate:  ①Efficiency；  ②Precision in diagnosis  ③Facilitation of communication; higher educational status • Barriers:  Trust and privacy concerns |
| Pelly et al., 2023 | No specific restriction | Via video conferencing | • Facilitate:  ①may be more acceptable post-COVID-19 in senior adults.  ②They are hesitant to discuss sensitive health matters with people. • Barriers:  ①Distrust in technology.  ②Credibility; Safety Credibility.  ③Prefer interacting with a human rather than an AI.  ④Some are not interested in AI-enabled systems.  ⑤were not familiar with AI or its functions. |
| Musbahin et al, 2021 | No specific restriction | A nominal group technique | • Facilitate:  ①Faster health services, Greater accuracy in management；  ②AI systems available 24/7, reducing workforce burden, and equality in healthcare decision making. • Barriers:  Data cybersquatting, bias and quality of AI data, less human interaction, algorithm errors and responsibility, and limitation in technology. |
| Benrimoh et al., 2021 | an AI-powered CDSS | Structured interview | • Facilitate:  ①Knowledge and skills gained.  ②Participant satisfaction.  ③Potential impact on clinical practice • Barriers: not mentioned |
| Zhang, 2021 | using AI-based   technology to comprehend   radiology imaging data | Semi-structured interviews. | • Facilitate:  ①Comprehend their physician’s diagnosis.  ②Seek more personalized and actionable information.  ③Perceived as a useful tool in allowing an individual to prepare for their clinical visits. • Barriers:  ①Concerns about the quality of AI prediction.  ②Lack empathy.  ③Privacy concerns.  ④Expressed concerns about the inability of AI to understand emotional issues. |
| Gundlack, 2025 a | Diagnosis.  Treatment; process optimization | Semi-structured focus groups | • Facilitate:  ①It’s efficient and has flawless functioning.  ②its ability to process and provide a large data volume, and increased patient safety. • Barriers:  ①potential data security issues.  ②fear of errors based on medical staff relying too  much on AI. |
| Krisnan, 2025 | AI in diabetes care | Semi-structured interviews. | • Facilitate:  ①Increased diagnostic speed, greater objectivity, and accuracy. • Barriers:  ①A prominent aspect was the apprehension regarding the accuracy of AI predictions, with some expressing worry about potential errors or misinterpretations.  ②in addition, concerns were raised regarding the appropriate implementation of AI tools, including uncertainties about how to integrate them effectively into existing health‑care systems. |
| Trivedi, 2025 | Conversational AI support  program for patients with AF in the community | Semi-structured interviews | • Facilitate:  ①the credibility of receiving information from hospital-associated providers.  Achieve human-like interaction  The continuity of support has been improved.  Improve access to health-related information.  Frequent reminders and information push notifications. • Barriers:  ①Data poverty and contextual shifts.  ②Cost effectiveness and health equity. |
| Tursynbek, 2024 | Medical artificial intelligence | Semi-structured interviews | • Facilitate:  ①by adopting certified AI-driven medical devices and regulated AI algorithms can efficiently ensure patient safety.  ②AI can significantly enhance the efficiency and accuracy of patient diagnosis and treatment. • Barriers:  ①AI is applied in practice, emotional support and interpersonal connections might be lost;  ②Expressed concerns about the inability of AI to understand emotional issues. |
| Gundlack, 2025 b | German health care system | Semi-structured interviews | • Facilitate:  ①Clear and understandable instructions for AI applications and explanations of their purposes are significant "Value of writing".  ②Ensure transparency to understand the development of artificial intelligence systems, data processing and usage  The goal was confirmed as crucial.  ③It is believed that AI systems should be easy to operate. • Barriers:  ①Due to the lack of understandability in the decision-making process of AI.  ②There is concern that the high cost may put patients at a disadvantage.  ③It is pointed out that if the doctor is skeptical or opposed to the AI system, the participants’ acceptance of these applications will decline |
| Sebastian et al., 2023 | No specific restriction | Conducted 4 experiments in which we manipulated the communication strategy (ethos, pathos, and logos) using screenshots of advertisements for a product | • Facilitate:  High innovative patients • Barriers:  privacy concerns, trust (especially related to the accuracy, efficiency, and precision of health IT systems),perceived novelty value. |
| Zhou et al., 2022 | No specific restriction | The authors tested all hypotheses in two separate experiments. | • Facilitate: not mentioned • Barriers:  ①Inter-group anxiety.  ②Patients worry about whether there is a privacy leakage and other issues that would harm their rights.  ③Symbolic group threats failed to pass the mediation effect verification. |
| Chen, 2022 | AI robot | Questionnaire | Age, educational level, and family support influence. • Facilitate: not mentioned  • Barriers:  ①"Too expensive"  ②"Complex to use, or unable to use" |
| Aggarwal et al., 2021 | No specific restriction | Self-completed questionnaire | • Facilitate:  ①Public trust and support;  ②Comfort with sharing data;  ③Regulatory guidance • Barriers:  ①Lack of awareness;  ②Due to privacy fears and anxiety;  ③Trust issues;  ④Data re-identification risks. |
| Armero et al., 2022 | No specific restriction | Questionnaire | • Facilitate:  ①Higher education levels.  ②Most patients believe the benefits of AI outweigh the risks.  ③AI as a significant advancement in medicine. • Barriers:  ①Lack of human qualities and low trust in technology.  ②Concerns about the security of protected health information.  ③AI-Cautious patients preferring the human nature of physician interaction.  ④Lack of awareness of how AI works in daily life. |
| Ayad et al., 2023 | AI in dentistry | Questionnaire | • Facilitate:  ①Improved diagnostic confidence.  ②Time reduction.  ③More personalized and evidence-based disease management. • Barriers:  ①The impact on workforce needs.  ②New challenges on doctor–patient relationships.  Increased dental care costs. |
| Baghdadi et al., 2024 | Use of AI as a Diagnostic Tool in Radiology | Questionnaire | • Facilitate:  ①Believe that AI might enhance the accuracy of radio-logical diagnosis.  ②People who are well educated in a certain subject tend to build more trust in it.  ③Excellent health status have also expressed a higher need • Barriers:  Divorced or widowed participants had a higher level of distrust of AI use in radiology. |
| Bahadir et al., 2024 | use of AI in dental radio graphic detection of occlusion caries. | Questionnaires | with higher educational levels • Facilitate:  AI-based diagnosis helped patients understand, believe, and trust their dentist's radiography. • Barriers:  Lack of trust |
| Esin et al., 2024 | No specific restriction | Questionnaires | • Facilitate:  ①Patients showed high trust in AI and robots in areas that go beyond the laboratory, such as radiology, pathology, and biochemistry.  ②This is believed to be due to the already existing use of computer systems in these fields. • Barriers:  ①High cost  ②Lack of human touch |
| Esmaeilzadeh et al., 2021 | No specific restriction | Designed a 2×3 experiment that crossed a type of health condition (ie, acute or chronic) with three different types of clinical encounters. | • Facilitate:  AI can improve health care delivery such as diagnostics, prognosis, and patient management • Barriers:  ①Incompatibility with instrumental, technical, ethical, or regulatory.  ②Perceived communication barriers.  ③Perceived transparency of regulatory standards.  ④Perceived liability issues.  ⑥Perceived trust in AI mechanisms, collaborative intelligence, and physicians.  ⑦Perceived privacy concerns.  ⑧Perceived social biases; perceived performance risks. |
| Fransen et al., 2024 | use of artificial intelligence in prostate cancer diagnosis on MRI | Questionnaires | Educational level • Facilitate:  ①Computer-generated second opinion; participants’ trust depended on the radiologists.  ②Influenced by the rapid development and increasing visibility of AI algorithms, • Barriers:  Accountability for AI |
| Huang et al., 2024 | AI-novice combination echocardiogram | Questionnaire | • Facilitate:  ①Accuracy､  ②Promote communication ③Reliability､  ④Recommendation from medical staff; ⑤Trust in doctors (knowledge and technology) ⑥There are ways to learn technology.  ⑦Awareness of AI technology • Barriers: not mentioned |
| Jutzi et al., 2020 | AI in Skin Cancer Diagnostics | A web-based questionnaire | • Facilitate:  ①Increase quality.  ②Ensure objectivity of skin cancer diagnostics.  ③Increase the accuracy of the algorithm relative to physicians. • Barriers:  ①Misused to their disadvantage by health and other insurance companies or employers.  ②Physicians might be tempted to rely on the AI-based algorithm.  ③With no personal conversation with the physician and less time for questions. |
| Kawsar et al., 2023 | AI as a medical device in  a skin cancer pathway | Online questionnaire | • Facilitate:  ①Saves time.  ②Doctor by analyzing photographs of my lesions • Barriers:  ①Feel uncomfortable.  ②Embarrassing having my photographs taken |
| Khullar et al., 2022 | No specific restriction | Using a hybrid probability-based, nationally representative online panel, | • Facilitate:  ①Reading your chest x-ray;  ②Making the diagnosis of pneumonia; • Barriers:  ①Misdiagnosis.  ②Privacy breaches.  ③Less time with clinicians.  ④Higher health care costs |
| Kosan et al., 2022 | AI in Dentistry | Questionnaires | • Facilitate:  ①Life easier.  ②Improves people’s quality of life.  ③Support dentists’ diagnostics in the future.  ④Prevent incorrect treatment.  ⑤Serve as a quality control for the dentist’s diagnosis; • Barriers:  ①Security concerns.  ②Scared of the topic and its consequences.  ③Fear that AI can make mistakes and lead to wrong decisions by the dentist. |
| Lennartz et al., 2021 | AI in Patients Across the Medical Workflow. | Single-center questionnaire | • Facilitate:  ①Cautious optimism.  ②Transparency and explicability.  ③Planning treatment according to recent state of science. • Barriers:  Severity medical conditions. |
| Liu et al., 2021 | No specific restriction | Questionnaire. | • Facilitate:  ①Shorter outpatient waiting time.  ②Accuracy rate.  ③Lower diagnosis expense.  ④Greater possibility to follow up after diagnosis • Barriers: not mentioned |
| Mahlknecht et al., 2023 | No specific restriction | Questionnaires | • Facilitate:  ①Save time and medical visits.  ②Rapid and intelligible use • Barriers:  Impeding and require additional time and effort of the GP's. |
| Meyer et al., 2020 | Usefulness of an Artificial Intelligence–Assisted Symptom Checker | Online questionnaires | • Facilitate:  ①Better understand what could be causing my symptoms.  ②Help me determine where I should seek care.  ③Better understand the diagnosis.  ④Get medical advice; • Barriers: not mentioned |
| Riedl et al., 2024 | use AI in cardiology, orthopedics, dermatology, and psychiatry. | Based on this 3 × 4 experimental within-subjects design. | • Facilitate:  trust, treatment adherence, and satisfaction. • Barriers:  ①Distrust and perceived privacy invasion.  ②Patients prefer direct face-to-face communication in their interaction with doctors.  ③Concerns about confidentiality |
| Yang, 2019 | No specific restriction | Questionnaire | Participants’ gender, race, treatment received and AIM related knowledge might influence their attitudes toward the AIM. • Facilitate:  Economical and convenient. • Barriers:  Lack of capability to deal with complicated disorders. |
| Parry et al., 2023 | Use of AI in Orthopedics | Questionnaire | Orthopedic surgery patients on average appear comfort able with the use of AI in their care. A patient’s overall comfort level appears to be influenced by age, education level, knowledge of AI, and perceptions of the effects of the technology on clinical outcomes and healthcare costs. • Facilitate: • Barriers:  Patients do not appear comfortable with autonomous AI-driven surgical robots |
| Ongena et al., 2020 | No specific restriction | Questionnaires | • Facilitate:  ①Have a good understanding.  ②Be able to ask questions personally.  ③Provides as much information; • Barriers:  Distrust and accountability; |
| Temple et al., 2023 | The implementation of AI in radiology. | Questionnaires | • Facilitate:  Efficiency of AI in radiotherapy: • Barriers:  ①Distrust and accountability.  ②Lack of personal interaction with ai in radiotherapy; |
| Alsanosi, 2025 | healthcare systems | Questionnaires | • Facilitate:  ①The understanding and familiarity with technology will promote acceptance.  ②Their stronger adaptability and lower skepticism towards privacy issues. • Barriers:  ①Data limitations, algorithmic biases, and the multi-factorial nature of human behavior;  ②Artificial Intelligence often faces challenges such as incomplete data, generalization errors, and a lack of transparency in the decision-making process; |
| Bersu Ozcan, 2025 | Artificial intelligence in screening mammography | Questionnaires | • Facilitate:  Efficiency of AI in radiotherapy: • Barriers:  ①There is a lack of interpersonal interaction concerns about loss, privacy leakage, insufficient transparency and risk of bias. |
| Tirapelli, 2025 | Artificial intelligence in dental  imaging diagnostics | Questionnaires | • Facilitate:  ①patients are aware that AI can make processes faster and more accurate in image processing diagnosis. • Barriers:  ①Data poverty and contextual shifts.  ②Cost effectiveness and health equity.  ③New technological colonization and potential exploitation.  ④Age and educational level is that centers with younger and more educated participants were more skeptical about AI’s potential to surpass human dentists. |
| Chan, 2025 | Artificial intelligence and  machine learning in cancer care. | Questionnaires | • Facilitate:  ①The patient is willing to support the application of AI/ML in critical tasks. It mainly stems from a high level of trust in oncologists rather than in the AI/ML technology itself. • Barriers:  ①Data privacy.  ②Lack of personal interaction with ai in radiotherapy; |
| Fu et al., 2023 | virtual reality [1] technology | Self-designed questionnaire | • Facilitate:  ①Experienced in interacting with humans and high-tech equipment;  ②The system is easy to learn and use, has strong applicability, and helps patients improve their training compliance. • Barriers: not mentioned |
| Liu et al., 2019 | No specific restriction | Self-designed questionnaire | • Facilitate:  Cognitive benefits • Barriers:  Patients lack a deep understanding of AI medical care, and have concerns and doubts about its safety, ease of use and communication ability |
| Zhang et al., 2022 | Pai Bao P2 robot is used for health education. | Self-designed questionnaire | Younger patients, those with higher levels of education, as well as their families and healthcare workers, have a higher acceptance rate for health education conducted by robots. • Facilitate:  ①Convenient anytime, anywhere.  ②Simple and fast operation.  ③Novel and interesting.  ④Reduces droplet transmission.  ⑤Rich content, diverse functions.  ⑥Reduces the workload of medical staff.  ⑦Can accompany me and move with me.  ⑧Robots don't get tired, can be reused, and don't have to worry about inconveniencing others. • Barriers:  ①Don't know how to use it, it feels too complicated.  ②The tone is stiff and rigid.  ③The robot is too cold, lacks emotion.  ④Not sensitive enough, can't understand my speech well.  ⑤Answers questions not personalized enough.  ⑥The battery drains quickly.  ⑦Feels unreliable, lacks a sense of security.  ⑧Too much hassle, not as convenient as talking to a person directly. |
| Zhu et al., 2019 | hospital intelligent medical system | Questionnaire | • Facilitate:  ①Self-efficacy.  ②To sense the system's usefulness.  ③Sensing system ease of use. • Barriers: not mentioned |
| Haggenmuller et al., 2024 | No specific restriction | Questionnaire | • Facilitate:  ①Diagnostic accuracy and explainability.  ②Participants preferred an increased explainability with display of both decision criteria and relevant image regions. • Barriers:  If they did not provide explanations on a case-by-case basis. |
| Macri et al., 2024 | AI-Generated Presenter | Questionnaire | • Facilitate:  ①Understood what the presenter was saying.  ②Felt at ease with the presenter.  ③Trusted this presenter. • Barriers:  The unknown about AI, Information on where to obtain commercial equipment to assist with posturing” |
| van der Zander et al., 2022 | No specific restriction | Questionnaire | • Facilitate:  ①Higher level of education.  ②Time saving.  ③Faster diagnostics and shorter waiting times • Barriers:  ①The most important disadvantage for GI-patients was the potential loss of personal contact.  ②Uncertainty about laws and regulations |
| Katirai et al., 2023 | No specific restriction | Conducted a two-part, exploratory workshop | • Facilitate: Improved hospital administration; Improved quality of care; Positive changes in roles and relationships; Cost reductions; Better patient experience; Reducing disparities. • Barriers: Concerns about changes to healthcare; Limitations and loss of autonomy; Technical issues and accountability; Emerging disparities; Data management issues; Costs of implementation. |
| Gonzalez et al., 2024 | ML-CDSS | A sequential explanatory study was conducted. | • Facilitate:  ①Rapid development of ML-augmented tools.  ②Trust in perioperative ML-CDSS depended on its role as a supplementary tool for the perioperative team.  ③Enhance their understanding of their medical condition.  ④Confidence in the positive impact of ML-CDSS on the quality of care • Barriers:  ①Using phrases such as “machine learning” and “AI” to describe ML-CDSS, which can have negative connotations for the public.  ②Overriding traditional clinician training approaches.  costs related to perioperative ML-CDSS should be transparent and included in insurance coverage.  ③Uncertainty about the process to verify data used for ML-CDSS development.  ④The accuracy of patient-reported data.  ⑤Diverse digital and health literacy levels, along with overwhelmed mindsets.  ⑥Concerns with the ability of ML-CDSS to make rapid and accurate recommendations in emergency or rare surgical situations.  ⑦Accentuate healthcare biases. |
| Palmisciano et al., 2020 | AI in Neurosurgery | A 2-stage cross-sectional survey, a qualitative survey and a case-based quantitative survey | • Facilitate:  ①Accuracy, communication, and confidentiality.  ②Acquired, interpreted  ③Improve diagnostic workflow; • Barriers:  Feel a lack of emotional support |
| Robertson et al., 2023 | AI in diagnosis | Qualitative study per-tested the vignettes and generated hypotheses quantitative phase as a blinded, randomized survey experiment | • Facilitate:  ①The patient toward AI as the established option.  ②Education level.  ③Age.  ④Generalized trust in AI companies; • Barriers:  ①Ethnic and racial differences.  ②Perceived lack of personalization.  ③Distrust in AI; |
| Witkowski et al., 2024 | No specific restriction | A web-based survey of 600 us-based adults | • Facilitate:  ①To support doctors within the therapeutic alliance.  ②Noted AI’s ability to serve as a workload easier.  ③Younger.  ④High educational.  ⑤Higher rates of decision self-efficacy.  ⑥Easing the workload with simple tasks like diagnostics.  • Barriers:  ①Didn’t "trust" AI.  ②Uncomfortable.  ③AI could not provide a “human touch.  ④AI lacked empathy, judgment, respect, close contact. |

Note: AI: artificial intelligence; ML-CDSS: Machine Learning-Clinical Decision Support System; GI: gastrointestinal ; COVID: Corona Virus Disease 2019; PwPD: Persons with Parkinson's Disease; RMS: remote patient monitoring solutions ;T2D:type 2 diabetes; ICD: implantable cardiovascular-defibrillator; SCD: sudden cardiac death; GP: General practitioners; CC: Cervical cancer; PHMI: people with a history of myocardial infarction; WTA: willingness to accept; DCE: Discrete Choice Experimen

**Table S7.** Quality assessment of included studies using the mixed-methods appraisal tool.

| Author(s),year | Screening | | Quality terms | | | | | Score |
| --- | --- | --- | --- | --- | --- | --- | --- | --- |
| Qualitative | S1 | S2 | 1.1 | 1.2 | 1.3 | 1.4 | 1.5 |  |
| Haan et al., 2019 | YES | YES | 1 | 1 | 1 | 1 | 1 | 5***** |
| Jalil et al., 2019 | YES | YES | 1 | 1 | 1 | 1 | 1 | 5***** |
| Adams et al., 2020 | YES | YES | 1 | 1 | 1 | 1 | 1 | 5***** |
| Nelson et al., 2020 | YES | YES | 1 | 1 | 1 | 1 | 1 | 5***** |
| Musbahi et al, 2021 | YES | YES | 1 | 1 | 1 | 1 | 1 | 5***** |
| Zhang, 2021 | YES | YES | 1 | 1 | 1 | 1 | 1 | 5***** |
| Wang et al., 2023 | YES | YES | 1 | 1 | 1 | 1 | 1 | 5***** |
| Godoy Junior et al., 2024 | YES | YES | 1 | 1 | 1 | 1 | 1 | 5***** |
| Mikkelsen et al., 2023 | YES | YES | 1 | 1 | 1 | 1 | 1 | 5***** |
| Pelayo et al., 2023 | YES | YES | 1 | 1 | 1 | 1 | 1 | 5***** |
| Robinson et al., 2023 | YES | YES | 1 | 1 | 1 | 1 | 1 | 5***** |
| Pelly et al., 2023 | YES | YES | 1 | 1 | 1 | 1 | 1 | 5***** |
| Maris et al., 2024 | YES | YES | 1 | 1 | 1 | 1 | 1 | 5***** |
| Sachdeva et al., 2024 | YES | YES | 1 | 1 | 1 | 1 | 1 | 5***** |
| Tursynbek, 2024 | YES | YES | 1 | 1 | 1 | 1 | 1 | 5***** |
| Krisnan, 2025 | YES | YES | 1 | 1 | 1 | 1 | 1 | 5***** |
| Benrimoh et al., 2021 | YES | YES | 1 | 1 | 1 | 1 | 1 | 5***** |
| Trivedi, 2025 | YES | YES | 1 | 1 | 1 | 1 | 1 | 5***** |
| Gundlack, 2025 a | YES | YES | 1 | 1 | 1 | 1 | 1 | 5***** |
| Gundlack, 2025 b | YES | YES | 1 | 1 | 1 | 1 | 1 | 5***** |
| **Quantitative RCT** | S1 | S2 | 2.1 | 2.2 | 2.3 | 2.4 | 2.5 |  |
| Zhou et al., 2022 | YES | YES | 0 | 1 | 1 | 0 | 1 | 3*** |
| Sebastian et al., 2023 | YES | YES | 1 | 1 | 1 | 1 | 1 | 5***** |
| **Quantitative non-randomized** | S1 | S2 | 3.1 | 3.2 | 3.3 | 3.4 | 3.5 |  |
| Chen, 2022 | YES | YES | 1 | 1 | 1 | 1 | 1 | 5***** |
| Aggarwal et al., 2021 | YES | YES | 1 | 1 | 1 | 1 | 1 | 5***** |
| Armero et al., 2022 | YES | YES | 1 | 0 | 1 | 1 | 1 | 4**** |
| Ayad et al., 2023 | YES | YES | 1 | 1 | 1 | 0 | 1 | 4**** |
| Baghdadi et al., 2024 | YES | YES | 1 | 1 | 1 | 1 | 1 | 5***** |
| Bahadir et al., 2024 | YES | YES | 1 | 1 | 1 | 1 | 1 | 5***** |
| Esin et al., 2024 | YES | YES | 1 | 1 | 1 | 0 | 1 | 4**** |
| Esmaeilzadeh et al., 2021 | YES | YES | 1 | 1 | 1 | 1 | 1 | 5***** |
| Fransen et al., 2024 | YES | YES | 1 | 1 | 1 | 1 | 1 | 5***** |
| Huang et al., 2024 | YES | YES | 1 | 1 | 1 | 1 | 1 | 5***** |
| Jutzi et al., 2020 | YES | YES | 1 | 0 | 1 | 1 | 1 | 4**** |
| Kawsar et al., 2023 | YES | YES | 1 | 0 | 1 | 0 | 1 | 3*** |
| Khullar et al., 2022 | YES | YES | 0 | 1 | 1 | 1 | 1 | 4**** |
| Kosan et al., 2022 | YES | YES | 1 | 1 | 1 | 1 | 1 | 5***** |
| Lennartz et al., 2021 | YES | YES | 0 | 1 | 1 | 0 | 1 | 3*** |
| Liu et al., 2021 | YES | YES | 0 | 1 | 0 | 1 | 0 | 2** |
| Mahl Knecht et al., 2023 | YES | YES | 1 | 1 | 0 | 1 | 1 | 4**** |
| Meyer et al., 2020 | YES | YES | 0 | 1 | 1 | 0 | 1 | 3*** |
| Riedl et al., 2024 | YES | YES | 1 | 1 | 1 | 1 | 1 | 5***** |
| Yang, 2019 | YES | YES | 1 | 1 | 1 | 0 | 1 | 4**** |
| Temple et al., 2023 | YES | YES | 1 | 1 | 1 | 0 | 1 | 4**** |
| Parry et al., 2023 | YES | YES | 1 | 0 | 1 | 1 | 1 | 4**** |
| Alsanosi, 2025 | YES | YES | 1 | 1 | 1 | 1 | 1 | 5***** |
| Bersu Ozcan, 2025 | YES | YES | 1 | 1 | 1 | 1 | 1 | 5***** |
| Tirapelli, 2025 | YES | YES | 1 | 1 | 1 | 1 | 1 | 5***** |
| Ongena et al., 2020 | YES | YES | 1 | 1 | 1 | 1 | 1 | 5***** |
| Chan, 2025 | YES | YES | 1 | 0 | 1 | 1 | 1 | 4**** |
| **Quantitative descriptive** | S1 | S2 | 4.1 | 4.2 | 4.3 | 4.4 | 4.5 |  |
| Fu et al., 2023 | YES | YES | 1 | 1 | 1 | 1 | 1 | 5***** |
| Liu et al., 2019 | YES | YES | 0 | 0 | 0 | 1 | 0 | 1* |
| Zhang et al., 2022 | YES | YES | 0 | 1 | 0 | 1 | 1 | 3*** |
| Zhu et al., 2019 | YES | YES | 1 | 0 | 1 | 1 | 1 | 4**** |
| Macri et al., 2024 | YES | YES | 1 | 1 | 1 | 1 | 0 | 4**** |
| Haggen muller et al., 2024 | YES | YES | 0 | 1 | 1 | 0 | 1 | 5***** |
| **Mixed methods** | S1 | S2 | 5.1 | 5.2 | 5.3 | 5.4 | 5.5 |  |
| Gonzalez et al., 2024 | YES | YES | 1 | 1 | 1 | 1 | 1 | 5***** |
| Palmisciano et al., 2020 | YES | YES | 1 | 1 | 1 | 1 | 1 | 5***** |
| van der Zander et al., 2022 | YES | YES | 1 | 1 | 0 | 0 | 1 | 3*** |
| Robertson et al., 2023 | YES | YES | 1 | 1 | 1 | 1 | 1 | 5***** |
| Katirai et al., 2023 | YES | YES | 1 | 1 | 1 | 1 | 1 | 5***** |
| Witkowski et al., 2024 | YES | YES | 1 | 1 | 1 | 1 | 0 | 4**** |

**Table S8.** The corresponding text of obstacles and promoting factors.

|  | Example quote(s)，(Author and year) |
| --- | --- |
| Lack of Humanistic Care in Artificial Intelligence | "Human Connection. Some participants were concerned about the lack of human connection that AI may imply and emphasized the need for “human empathy” and the “ability to understand with flexibility”.[2] "The notion that AI might overshadow human interactions presents a barrier to its widespread acceptance."[3] "Patients express their concerns about depersonalized procedures in which patients become numbers."[4] "Worries about the use of AI in general practice. A worry regarding AI among six of the interviewees, three men and three women, concerned AI taking over the GP's position and the patients losing their relationship with the GP."[5]  "In the realm of emotion, patients noted AI’s lack of compassion and empathy. One patient expressed, “You can’t write an algorithm to love somebody.” In the realm of non-verbal communication, patients called attention to AI’s lack of emotion perception, “eye contact,” and “human touch."[6] "Losing the emotional side of patient-doctor relationships."[1] "AI-based communication often lacks intentional and therefore constitutes a significant obstacle at the communication level."[7] "Surgeons generally have concerns about approaches that lack the sense of touch in surgical procedures."[8]  "Reliance on AI clinical applications may reduce physicians’ and patients’ interactions and conversations. Consumers may refuse to use AI applications because they need human social interaction during service encounters."[9] "Another common concern regarding the use of AI was a diminished physician-patient relationship—consultations could become more sterile, with no personal conversation with the physician and less time for questions."[10]  "However, the patients who discussed the findings with their physicians conveyed mixed experiences about whether physicians were interested or open about discussing symptom checker results."[11]  "The diagnosis and treatment of psychiatric disorders, which are often not discussed openly... are based more on the direct interaction between doctor and patient (i.e., human-human interaction) than the diagnosis and treatment of illnesses in other medical disciplines."[12] "On the other hand, 29.5% of the patients indicated not using the symptom checker at home, mostly due to a preference for personal contact with the GP or because they saw no additional benefit of a symptom checker."[13] "The lack of humane care was regarded as one defect of AI doctors."[14] "The most important disadvantage for GI-patients was the potential loss of personal contact with healthcare professionals ."[15] "Impact on Personal Interaction: "45% of participants felt that using the application made the interaction with patients feel less personal or that it interfered with their interview."[16] "Fear of losing the ‘human touch’ associated with doctors was a common theme within qualitative coding, suggesting a potential conflict between the implementation of AI and patient-centered care."[17] "Patients desire significant personal interaction with healthcare professionals during the course of their treatment."[18] "For serious topics like cancer or a disease that may be killing you, you don’t want AI telling you ‘you’re going to die’. [. . .] we are not at the point where AI basically understands emotions. So in kind of sensitive things, you kind of just want it to be like, ‘hey, here’s your results, here are the steps you can do while you’re waiting for your doctor.’"[19] |
| Insufficient Patient Privacy Security Protection | "They expressed concerns that such recordings could inadvertently capture intimate moments, potentially violating their privacy and that of family members, housemates, and friends."[3] "The concerns seemed to come from the interviewees fear of the above-mentioned misuse of their data."[5]  "Privacy and data protection concerns were a significant point of discussion in each of the six FGs and different perspectives were mentioned. Less than a third of participants, especially smartphone users, feared that their images could potentially be published on social media pages, like Facebook."[20] "Some expressed concern about the privacy of information fed to the AI system."[21] "Issue of privacy and where any health information artificial intelligence systems are kept."[1]  "We also identified that patients were less willing to share data with commercial organizations. Privacy fears and anxiety that the transferred data may be used for profit could explain this finding."[22] "Patients in the AI-cautious group reported an increased concern for the risk of protected health information security breaches and a lack of trust in technology compared to the pro-AI group."[2] "The first is the belief that anonymize data can be identified through AI models, and in turn, could increase the likelihood of privacy invasion and data breach. The second is that AI systems need massive data sets; thus, patients are concerned that their health information may be collected or shared without permission for purposes other than treatment."[9] "The concerns most frequently voiced in this survey were related to data protection, impersonation and susceptibility to errors."[10] "Patients fear data breaches in the doctor with AI system and in the AI system only conditions more than in the human doctor condition."[12] "Data privacy concerns can hinder technology adoption,particularly when many data privacy directives."[23] "Patients are concerned that AI medical devices may malfunction, leak privacy, or have other risks due to technical deficiencies, program vulnerabilities, or poor management."[24] "Patients are concerned that smart health systems may cause equipment failures, privacy breaches, or other risks due to inadequate technology, procedural vulnerabilities, or poor management."[25] "One of the reasons for this bias and resistance is from realistic individual threats; that is, patients worry about whether there is a privacy leakage and other issues that would harm their rights."[26] "It’s just those concerns that how safe it is out there. Well, you hear things get hacked. What happens if my health stuff gets hacked and it can be used against me?"[19] |
| Uncertainty about New Technology | "Fear of the Unknown. Many participant initial perceptions of AI were shaped by popular media and science fiction."[27] "Patients report that they are unsure about the skills of a computer. They value the experience of the radiologist."[4] "I would probably need…feedback from a medical professional to…trust the app,” stated one patient, “because it’s like a black box…Algorithms with databases behind them…can make errors."[6] "At first it was hard for me to kind of trust it a little bit, because I was so used to doing the finger stick. And I just wasn't sure what the technology, what was going to happen?"[28] "Distrust of AI was based on unknown reliability, scepticism of AI algorithms and generational scepticism."[21] This is a key finding; if the use of AI in healthcare is to increase,educating patients about the risks and benefits of this technology is crucial.[22] "Participants in scientific and administrative fields reported lower levels of distrust in AI compared with participants with no specialty (P<.001). People who are well educated in a certain subject tend to build more trust in it."[29] "Thirty-one percent of respondents reported being very uncomfortable and 40.5% were somewhat uncomfortable with receiving a diagnosis from an AI algorithm that was accurate 90% of the time but incapable of explaining its rationale."[30] "The conceptual propaganda for AI diagnosis and treatment are still not in place, particularly in some rural areas and among some old people with relatively traditional medical concepts."[24] "Less than half of the participants had ever heard of AIMs, which is less than that in the Central and Eastern Europe (CEE) population."[14] "The main reason for the gradient trust is that patients lack a deep understanding of AI medical treatment and are worried and suspicious about its safety, ease of use, and communication ability."[24] "Patients' understanding of the smart medical system mainly stays on the surface and lacks a deep understanding, which affects their trust in the system and willingness to use it."[25] "Patients’ experience with AI was significantly associated with average AI comfort level (p < 0.0001), with patients who responded that they do not know what the terms “AI” or “ML” mean or have unknown experience with AI/ML having the lowest average AI comfort level (mean: 5.6; SD: 2.6)."[31] "One third of patients was unfamiliar with AI, leaving room for better dissemination of information."[15] |
| Lack of Trust in Artificial Intelligence | "A significant number of participants voiced uncertainties about the accuracy of health insights generated by AI, and a prevailing unfamiliarity with AI was observed among both Pd and neurologists."[3] "when scientific research shows that computers are indeed superior to humans, most patients indicate that they would rather let the computer do the work than the radiologist."[4] "Lack of trust in AI was also reflected in one of the interviewees’ statements: “AI is not a living creature and depending on what you feed it with, it can learn different things, so it is important to be critical"[5] "Credibility (30 [63%]) was another common theme that emerged."[6] "Some participants felt that virtual care is inadequate when they believe a physical exam is needed to fully evaluate their medical problem. "[32] "Lack of complete trust in the diagnosis was often attributed to the fact that the system is not necessarily 100% error-free and can malfunction. Te competence of HCPs while filming using the smartphone was also evoked as a factor that could influence the accuracy of the diagnosis."[20] "Safety of advice provided over an AI-enabled chat bot was questioned by participants."[21] "Our findings add to a downward trend in public trust regarding sharing data with commercial organizations, which seems to have changed significantly when compared to historical evidence."[22] "In comparing the Pro-AI and AI-cautious groups (Figure 2D), the AI-cautious patients foresaw more risks in the implementation of AI, including the belief that the algorithms may not be as  good as physicians, highlighting a lack of trust in the technology or concern about the risks of loss of privacy of health information."[2] "Also assessing patients’ concerns about a topic they may not fully understand, such as the use of AI in diagnosing occlusion dental caries, could potentially lead to subjective results."[33] "However, it revealed that physicians have also concerns about potential medical errors and liability issues that AI may cause."[8] "Individuals with chronic conditions may not trust AI clinical applications if no physician interactions are included in health care delivery. [...] The nature of AI models (such as deep learning) may increase a lack of transparency related to AI systems and threaten patient trust, resulting in higher risk beliefs."[9] "Patients favored physicians over AI for all clinical tasks except for treatment planning based on current scientific evidence."[34] "Despite ongoing concerns about symptom checker accuracy, a large patient-user group perceived an AI-assisted symptom checker as useful for diagnosis."[11] "AI adoption has established that technology trust influenced the behavioral intention of the use of AI products."[23] "The therapeutic capacities of AI still do not appear to be entirely proved, which might be why knowledge reserve was not one of the factors influencing people’s trust in the therapeutic advice made by an AI doctor independently."[14] "Patients doubt whether AI can communicate smoothly with them, understand their pain, and provide personalized treatment plans based on their conditions."[24] "When the patient makes the corresponding action according to the instructions of the robot, no one is beside to correct it, and they do not know whether it is correct. The robot feels insecure and insecure."[35, 36] "Concerns about ML-CDSS’s ability to make predictions in emergency and rare medical situations."[37] ""42% of patients (95% CI: 34%-49%) found AI systems unacceptable if neither the dermatologist nor the patient could trace the assessment."[38] "Overall, patients were moderately negative on the subject of distrust and accountability, with an average score of 3.28. This indicates that patients have concerns about AI taking over diagnostic interpretation tasks of the radiologist, both with regard to accuracy, communication, and confidentiality."[39] "I would need proof that it works and what you’re actually getting is meaningful information. Like it’s not just some crap. If it’s going to make recommendations to me, I want them to be proven that they’re actually legit."[19] |
| Public Fear and Resistance to New Technology | “ear of experiencing new things respondents expressed fear of robot rehabilitation training.”[40] |
| Reduced Self-efficacy | "The second largest cluster of concerns was the perceived limitations of AI and potential loss of autonomy for both HCPs and patients. There was an implicit assumption that the introduction of AI into healthcare would require direct communication between patients and robots or other AI-powered entities, to which care would be delegated."[41] |
| Imperfect Artificial Intelligence Technology | "Lack of Wireless Capability :The device only functioned with wired internet that had to be connected through a cable through the telephone port in a patient’s house."[42] "Several participants expressed that they expected that the sheer amount of data associated with AI-driven technologies would hinder transparency. They emphasized the ongoing importance of trust and confidence in doctors."[43] "No guidelines or framework to monitor the creation of artificial intelligence algorithms"[1] "The GPs, who together with their medical assistants, perceived the entirety of the various patients’ difficulties in using the symptom checker, strongly agreed to recommend the symptom checker mostly for younger patients and those with skills in using digital devices."[13] "For GI-physicians, the most important disadvantage was insufficiently developed IT infrastructures."[15] |
| Poor New Technology Usage Experience | 训练中的生理不适感 脑卒中患者患病后常伴肌力下降和肌张力异常,易产生疲劳感,这可能会导致训练时间减少.[44] "Every patient criticized the galvanometer. It was difficult to use. It also gave uncomfortable experiences."[42] "One participant expressed particular concern about how healthcare workers perceive and use these technologies, both in terms of their expectations and actual implementation."[43] "Participants expressed their belief that doctors are the most knowledgeable as to which type of screening would be most appropriate for an individual patient and emphasized their trust in the expertise of health care professionals."[32]  "I trusted everything because of my doctor. It took me the first day [to trust the device] I just trusted."[28] "Participants believed credible sources of information and recommendations from the healthcare team are important to develop trust."[21] "Their results showed that participants’ trust depended not only on AI technology but also on the radiologists, whom they trusted to utilize thoroughly tested, beneficial tools."[45] "The robot's answers are too stiff and inflexible, the speed of speech is a little fast, and if the question is not accurately asked, or the Mandarin is not standard, the robot may not be able to recognize and give accurate answers."[36] |
| Insufficient Data Reliability and Transparency | "participants expressed safety concerns that computers could make serious mistakes, and some noted uncertainty regarding the accuracy of AI."[32] “Patients were significantly more concerned about the transparency of regulatory standards to assess AI algorithms and tools in comparison with the transparency of guidelines to monitor the performance of physicians’ practices for both acute and chronic conditions.”[9] "Further perceived problems that were mentioned were the non-traceability of the decision algorithms and the missing transparency of the applied systems."[10] "The use of AI in medicine without adequate disclosure or explanation to patients can be hazardous.transparency and excitability are absolutely crucial for AI implementation."[34] |
| Cybersecurity Not Ensured | "Hacking and cybersquatting of health data."[1] "This would reduce their ability to classify lesions without the assistance system as well as their ability to notice obvious mistakes or malfunctioning of the algorithm itself, which could occur due to various technical problems or even deliberate manipulation by hackers."[10] |
| Unclear Medical Responsibility Attribution | "Accountability is related to the responsibility of humans when computers make mistakes. Patients note that radiologists can be held accountable for their mistakes, and they wonder who can be held responsible for errors made by computers."[4] "Ultimately, only a second human doctor would be able to bear the moral responsibility a second opinion holds."[43] "Who is responsible if AI produces a bad health outcome?"[1] "Patients with acute conditions were more likely to be concerned about liability issues [...] due to the lack of clarity about who is responsible if AI-recommended treatment options are mistakenly dismissed or offer wrong recommendations."[9] "The study further emphasized that in instances of misdiagnosis, participants deemed the hospital, radiologist, and AI program developer accountable, in descending order of accountability."[45] "Uncertainty about laws and regulations (responsibility) was a significant concern for both GI-patients (48.5%, n = 166) and GI-physicians (35.0%, n = 28)."[15] |
| High Cost of New Technology Application | "AI-based dental care will raise new questions about cost-effectiveness and ever rising healthcare costs."[7] "In addition, the risk of unequal opportunities due to potential high costs not covered by standard health insurance was pointed out."[10] "Some old people would rely on their own self-healing function or immune system rather than go to the hospital due to their outdated concepts of affordably diagnosis and treatment expenses."[46] "17.71% of respondents are concerned about the high cost of AI medical treatment."[47] |
| Cultural Adaptability and Inter-group Threat | "Cultural Acceptability. Some participants expressed concern that AI is at odds with cultural ways of knowing and healing."[27] |
| May Make Patients Unwilling to Persist in Using New Medical Technology to Manage Their Health | "I may be less likely to jump into new technology because I have the other technology i can compare it to. So, I'm using older information like we have our fingertip cyclometers that we can check and compare to our readings. So, if there's a backup or something that you're familiar with to compare it to the new stuff, i think that makes it a lot easier to adapt."[28] |
| Facilitating Factors |  |
| Belief in AI's Faster Diagnostic Speed, High Efficiency, and Accurate Diagnostic Results | "There was a willingness to trust outputs from AI to obtain the most accurate information possible."[27] "They shared the belief that AI may be a helpful instrument to support medical decisions by gathering and summarizing patients’ clinical information, identifying patterns, and proposing best courses of action."[3] "proof of technology refers to the need of patients for high-quality studies that prove the value and reprehensibility of AI systems, which is the responsibility of the scientific community."[4] "Patients perceived more accurate diagnosis (33 [69%]) as the greatest strength of AI compared with human skin cancer screening."[6] "they believed that teleophthalmology represented more advanced technology that had greater precision and accuracy."[32]  "It is important to note that concerns regarding blind trust were voiced by a number of patients and providers in our study and that trust in the device was directly related to patient experience, device accuracy, and duration of device use."[28] "However, participants in all FGs underlined the usefulness of the application, especially in terms of increased efficiency, precision in diagnosis, and facilitation of communication."[20] "Trust in AI involves participants’ positive expectations of the outcome of AI. This category consists of five concepts: 1) Credibility, 2) Safety, 3) Distrust in technology, 4) Personification, and 5)Transparency."[21] "Benefits of Artificial Intelligence in Healthcare、Faster and quicker diagnosis reached by an AI system、Artificial intelligence algorithms can use the data to spot trends and patterns that humans are unable to determine."[1] "Previous studies have referred to AI as a facilitator of faster, more precise and more personalized and evidence based disease management. These studies have reported on par or even higher diagnostic accuracies than average dentists."[7] "patients were neutral in their trust of AI taking over radiologists’ diagnostic interpretation tasks; they believe that AI might enhance the accuracy of radio logical diagnosis."[29] "Our study found that the public was ready to undergo surgery performed by AI and robots, had trust in AI for follow-up care, and did not fear AI."[8] "Highlighting the performance benefits of AI, such as accuracy of diagnosis, reliability of data analysis, the efficiency of care planning, and consistency of treatments, in communication with users [...] may increase individuals’ intention to at least try services provided by AI applications in health care."[9] "they stated that the use of AI might lead to more reliable and less subjective diagnoses, which might lead to fewer unnecessary biopsies and less overlooked malignant lesions."[10] "The majority of our respondents felt confident in computers being used to help doctors diagnose and formulate management plans and as a support tool for general practitioners when assessing skin lesions."[48] "Patients assigned significantly higher mean scores to the physician rather than to AI for all capabilities included (Table 2), except for treatment planning based on the most recent scientific evidence, for which the participants favored AI to physicians"[34] "We found that ‘accuracy’ was the most important thing for respondents and most of the levels of attributes ‘diagnostic methods’ and ‘accuracy’ were statistically significant."[46] "Most patients thought the tool gave them useful information for their health problems (274/304, 90.1% either strongly agreeing or agreeing) with about half reporting positive health effects (154/302, 51.0%)."[11] "he most frequent patient-reported reasons for general satisfaction (free-text indications) were easy and rapid use of the symptom checker, precise and comprehensive questions, perceived time-saving potential, and encouragement of self-reflection."[13] "Most cancer patients trusted AIMs in both stages of diagnosis and treatment, and participants who had heard of AIMs were more likely to trust them in the diagnosis phase."[14] "Most respondents believed that AI medical treatment would bring benefits to patients (91.26%) and new problems (81.61%). The main benefits mentioned were: more convenient (77.13%), more efficient (70.4%), and more diagnostic methods (49.33%)."[47] " AI can provide expert-level accuracy in medical care."[26] "GI-patients (71.3%, n = 246) and GI-physicians (51.3%, n = 41) agreed that AI could lead to faster diagnostics and shorter waiting times."[15] "Time Constraints: "40% of participants felt the application would save them time, and 30% felt the application would neither cost nor save time."[16] "Confidence in ML-CDSS’s ability to improve surgical care outcomes. All patients indicated confidence in ML-CDSS’s ability to identify potential surgical complications from suggestive pat terns in patients’ medical data."[37] "Diagnostic accuracy was the most important AI feature in decision-making for patients, with an average importance of 21% (95% CI: 19%-22%)."[38] |
| Improved Patient-Doctor Communication | "Importance of in-person care for establishing an emotional connection with the doctor." "One participant specifically mentioned the importance of having support from human warmth and physical contact with their doctor, particularly during difficult times."[32] "Participants also recognized that the use of smart phones facilitated communication between providers, as well as patient-provider communication, since it allowed them to visualize their own cervix and any potential lesions after the gynecological exam."[20] "In addition, the preference for a human centered approach was also expressed in terms of the importance of patient-professional relationships, including empathy in communication."[45] |
| Positive Experience | "Therefore, making the process fun and pleasurable is important towards patient adoption."[49] "The robot also integrates chat function, voice greeting and entertainment function in one, which makes up for the lack of spiritual support for the patient's family and reduces the sense of loneliness."[36] |
| Protection of Patient Privacy (Sensitive Issues) | "Two health professionals agreed that some PHMI would use an AI chat-bot because they are hesitant to discuss sensitive health matters with people they know."[21] |
| Enhanced Self-efficacy | “Respondents said that receiving positive feedback from health care workers and robots during rehabilitation training helped boost confidence and self-fulfillment.”[40] "She believed that AI-driven clinical decision-making, based on hard data, could boost patient confidence in the doctor and the patient’s own assertion."[43] "This intuitively suggests that having the resources to improve access itself also improves overall patient experience and enjoyment, placing further importance on facilitating."[49] "Higher rates of decision self-efficacy were associated with greater confidence in the use of AI in medical care."[17] |
| Convenient Operation | "The system is easy to learn and easy to use, and has strong applicability, which helps patients to improve their training compliance."[50] "The robot is simple and convenient to operate, and the elderly can also operate easily."[36] "GI-patients appreciated the availability of AI at any time (24/7) (24.6%, n = 85) and remote communication (19.4%, n = 67)."[15] "AI video tools such as AI presenters may offer an opportunity to reduce the time and cost of creating a video.[51]" |
| Cost Savings | "Both GI-patients (18.0%, n = 62) and GI-physicians (16.3%, n = 13) mentioned cost savings as an advantage of AI in healthcare."[15] |
| Cybersecurity Ensured | "This indicates that when patients perceive that cybersquatting is ensured, it can facilitate their acceptance and use of AI in healthcare."[52] |
| Assist the doctor's decision-making function | "In addition, several participants believed that AI could mitigate human deficiencies, like stress and fatigue, that might otherwise negatively impact doctors’ judgments."[43] "Half of the interviewees expressed that they would even feel safer if the GP used AI as a support tool when diagnosing. This opinion was partly explained by the fact that GP's are quite busy and thus the risk of the GP's overlooking something important regarding patients’ health was perceived as a possibility that AI could prevent."[5] "Patients envisioned AI referring to a physician and providing a second opinion for a physician."[6] In addition, some participants believed that teleophthalmology "complements the eye exam per formed by a primary care clinician to catch diabetic eye disease that might otherwise be missed."[32] "Artificial intelligence can be used as a support tool to provide more information."[1] "However, as other studies have pointed out, AI will rather work synergistic with clinicians than replace clinicians completely by overtaking clinical work."[7] "The implementation of this technology can reduce the number of healthcare professionals required in these areas and streamline workflow."([8] "Patients were more willing to choose AI if the ultimate treatment decision rests with the physician and not only the AI."[9]  "Most patients preferred AI involvement alongside a radiologist in diagnosing PCA: 79% of the participants would like a second opinion by a computer program after a radiologist’s diagnosis, and 91% want a radiologist to have a second look after a computer diagnosis."[45] "This can be transferred to our scenario; trust in AI is the expectation that this technology will provide beneficial recommendations for a patient’s health, allowing for risks to be taken based on this expectation."[53] "“Patients were significantly more comfortable with the use of AI under the physician’s supervision than without such supervision."[34] "AI diagnosis acting in concert with clinicians will significantly guarantee the accuracy."[46] "We found that people prefer a human doctor, followed by a human doctor with an AI system, and an AI system alone came in last place."[12] "Most participants thought AIM would assist oncology physicians in the future, while little really believed that oncology physicians would completely be replaced."[14] "Especially during the COVID-19 epidemic, instructing patients to perform lung function exercises with coughing and deep breathing can cut off the transmission path of droplets and better protect the safety of medical staff."[36] "Participants mentioned AI’s ability to serve as a supportive data/evidence/tool for human healthcare providers, such as providing a second, third, or fourth opinion."[17] |
| Understanding the Importance of AI in Healthcare | "Procedural knowledge is related to the importance of understanding how AI will be implemented in the current radiological practice."[4] "Emphasized the importance of patient training and ready access to necessary information tools and resources."[28] "Pro-AI patients saw benefits from the introduction of AI, including advances in technology and medicine, as well as improved patient safety."[2] "Training should target the patient community to ensure that the patients obtain sufficient information to make informed health decisions. [...] If users understand the basics of AI applications, and the potential benefits and limitations they can bring to health care, they will become more willing to accept AI use."[9] "Educating patients about the benefits of the current healthcare system (e.g., increased diagnostic performance and productivity gain) might further persuade them toward this approach."[45] "The more patients understand VR technology through a variety of ways, the more likely they are to find that such technology has a certain effectiveness and practicality for their rehabilitation training."[50]  "All participants acknowledged the potential for preoperative ML-CDSS to enhance their understanding of their medical condition, thus facilitating shared decision making."[37] "It is important to demonstrate to patients that the use of AI in their healthcare is safe, effective, and ethical."[18] |
| Reliable Data (Fairness, Interpretability, Transparency) | "I want to make sure that that algorithm has been tested well in randomized control trials, to make sure that we're not getting."[28] "Some level of transparency would improve trust in AI. Participants expected reasoning behind the AI advice."[21] "Other reports highlight trust and open discussion as vital for the implementation of AI and other technologies."([2]  "Transparency of AI algorithms should be emphasized so that the AI system does not remain a black box to the users."[9]  "My level of trust would depend on the source naturally. If it’s from Joe down the street, obviously I wouldn’t be too crazy about it. But if it’s from a trusted source, like a well-respected medical organization or something like that, like John Hopkins or Mayo Clinic, that would probably help build a little bit of trust."[19] |
| Recommendations from Trusted Sources (Doctors) | "One participant expressed particular concern about how healthcare workers perceive and use these technologies, both in terms of their expectations and actual implementation."[43] "Participants expressed their belief that doctors are the most knowledgeable as to which type of screening would be most appropriate for an individual patient and emphasized their trust in the expertise of health care professionals."[32]  "I trusted everything because of my doctor. It took me the first day [to trust the device] I just trusted."[28] "Participants believed credible sources of information and recommendations from the healthcare team are important to develop trust."[21] "Their results showed that participants’ trust depended not only on AI technology but also on the radiologists, whom they trusted to utilize thoroughly tested, beneficial tools."[45] |
| Public Trust | "To ensure that the benefits of AI are secured in clinical practice, future research on best methods of physician incorporation and patient decision making is required."[54] |
|  |  |
| Severity of illness | "Participants who reported excellent health status have also expressed a higher need to obtain full disclosure and be informed by the AI diagnostic tool about their overall health status when compared with participants who reported their health status as average or fair/poor."[29] "The acceptance of AI was significantly lower for diseases of high severity (2.97 [SD 1.52]) than for diseases of medium severity ."[34] |
| Level of educational attainment | "Influence of educational status on the factors influencing the acceptance of AI‑based diagnostics As the FGs were organized according to the participant’s educational level, the influence of education on the previously described factors was explored. Most women of primary and superior levels of education saw the tool as an advanced use of technology and were curious about its use."[20] "Pro-AI patients with graduate education than AI-cautious patients, demonstrating that higher levels of education correlated with an increased understanding of AI and willingness to adopt its use."[2] "Regarding educational status, a statistically significant difference was observed only between participants with primary education and those with a high school education."[8] "Patients with higher education and brain-based work types have a higher acceptance of the system, generally such patients have a strong thirst for knowledge, a higher understanding ability, can get familiar with the process faster, will pay more attention to the disease situation, and have more opportunities to hear about or contact VR equipment or technology in life."[50] "Average AI comfort level also differed significantly by education (p = 0.0029), with patients with a graduate degree being most comfortable (mean: 6.9; SD: 2.1)."[31] "Factor 1 (distrust and accountability) was also significantly related to the education level of respondents; the level of trust steadily increased for each higher category in education level of respondents (F(4, 4) = 6.99, p < 0.01)."[39] |
| Age | “In terms of acceptance, young and educated patients, family members and medical staff have a higher acceptance of robot health education.”[36] "Average AI comfort level differed significantly between age groups (p = 0.032), with the 55– < 65 and 65– < 75 age groups having the highest average comfort level (mean: 6.8; SD: 2.4) and 18– < 45 and 45– < 55 having the lowest (mean: 6.0; SD: 2.4 and 2.3, respectively)."[31] "Factor 4 (efficiency) was weakly negatively associated with age (r = −0.200, p < 0.05), which means that the older the respondents are, the less they think that AI increases efficiency, while factor 2 (procedural knowledge) was weakly positively associated with age (r = 0.196, p < 0.05)."[39] "Older respondents had significantly lower odds of choosing AI (β = -0.065), suggesting that younger respondents are more amenable to AI-enabled interventions in their individual care."[17] |
| Family support | "Patients with family support disorders are vulnerable to obstruction by family members, and elderly patients living with their children or grandchildren often receive more publicity and education."[55] |
| Gender | \| "with females being less trusting of AI and more likely to desire human interaction (although a large male gender bias was present)."[18] \| \| --- \| |

Note：AI：artificial intelligence; ML-CDSS：Machine Learning-Clinical Decision Support System; GI：gastrointestinal; GP: General practitioners; HCPs: healthcare providers; FGs: focus groups

**Table S9.** Interventions from the included literature.

| Author(s),year | Intervening measure |
| --- | --- |
| Qualitative |  |
| Wang et al., 2023 | • Provide information support, eliminate patient resistance, optimize mechanical design, and alleviate physical discomfort experience. • Multiple interventions can be applied comprehensively to develop suitable and effective rehabilitation strategies for patients to improve their training compliance and motivation |
| Adams et al., 2020 | • Potentially through patient education campaigns; diagnostic accuracy and impact on health system processes |
| Godoy Junior et al., 2024 | • Provide tangible clinical benefits, remain user-friendly, and uphold trust within the physician-patient relationship. |
| Haan et al., 2019 | • The need for human interaction was unambiguously indicated. • The need to speed up the discussion of associated ethical issues and the development of legal AI regulations. |
| Jalil et al., 2019 | • The patients wanted to see their own data meaningfully presented through graphs. And a wireless device was preferred due to mobility. Glucometer comfort, inclusion of all medication names |
| Maris et al., 2024 | • Policies on patient-centered AI integration in clinical practice should encompass the ethics of everyday practice rather than only principle-based ethics |
| Mikkelsen et al., 2023 | • Transparency and Involvement; Education and Awareness; Ethical and Legal Frameworks; Patient-GP Collaboration |
| Nelson et al., 2020 | • Ensuring that AI tools are used in a manner that preserves the integrity of the human physician-patient relationship. Implementing transparent validation processes. Providing education |
| Pelayo et al., 2023 | • Leveraging primary care providers' recommendations; Human oversight of AI-based image interpretation; Improving communication |
| Robinson et al., 2023 | • Patients need to understand all information about AI and requires that all health care providers technology training. Patients also requested the ability to trial a number of devices and to be connected to all relevant systems to ensure that the device is appropriate for them. |
| Sachdeva et al., 2024 | • Ensure the protection of patients’ data and provide the patients with assurance regarding its strict conditionality. Convey the purpose, benefits, and potential risks of the tool in written form (brochures) for patients. |
| Pelly et al., 2023 | • Personalized advice from an AI-enabled system. • Some level of transparency would improve trust in AI • Provision of intelligent feedback |
| Musbahi et al, 2021 | • Inform future interventions or policymaking regarding the use of AI in healthcare |
| Benrimoh et al., 2021 | not mentioned |
| Zhang, 2021 | • The need to increase system transparency by explaining how AI arrived at its conclusion; Presenting the information sources underpinning a system; Providing personalized information is a key feature of intelligent and recommender systems;  • Participants generally expressed the desire to know the details about the system’s reliability, including the AI-prediction accuracy. |
| Gundlack, 2025 a | • Ensuring transparency in data usage and applications ‘goals  • Protecting data against misuse  • Clarify and define responsibilities before use  • Provide clear and brief explanations of AI's basic functions, recommendations and decisions  • Applications for patients should not include the need for medical knowledge  • Add different language functions, including simplified language  • Provide options and help for non-tech-savvy users  • Ensure high data quality  • Incorporating a human component into the treatment process  • Start with basic processes like structuring and offering information |
| Krisnan, 2025 | • Predictability ensures consistent AI performance, fostering user anticipation and comprehension. Dependability mitigates unforeseen consequences, while faith emerges when technology proves predictable and dependable, culminating in users fully trusting AI tools, nurturing positive relationships.  • Patients are more likely to accept an AI-assisted program if they receive some kind of guarantee about the accuracy of the AI system and that sensitive test results will still be delivered by human clinicians.  • Improved accuracy, reduced bias, and increased free time to interact with doctors as the primary benefits of AI. |
| Trivedi, 2025 | • Enhance the interactive flexibility of conversational AI calls to make conversations more natural and smooth.  • Enhance the personalization level of health scenarios.  • Customize the amount of information according to individual preferences.  • Reduce the repetition of content |
| Tursynbek, 2024 | • Popularizing the application of medical artificial intelligence among patients is beneficial for resolving their doubts. It is of great significance to consider and support medical staff in carrying out various diagnostic and therapeutic interventions and ensure ethical innovation.  • When integrating artificial intelligence, ethical, legal, and technical issues must be handled with caution, with a focus on protecting patients' privacy and ensuring openness.  • First of all, AI should be used in combination with human supervision. Although the patient understands and recognizes the advantages of AI, but remain cautious about its autonomous use.  • Secondly, medical staff should receive training on AI applications.  • Thirdly, clear ethical guidelines need to be established to ensure Patient privacy protection, data security, and transparency in AI usage.  • Finally, it should be implemented A regular evaluation mechanism is established to promote the continuous improvement of AI. |
| Gundlack, 2025 b | • The subjects believe that artificial intelligence systems should be tested like medical devices. Testing through external verification and long-term use will be of positive significance.  • Human resource support is needed.  • Suggested adoption ensures with adaptive interpretation, multilingual support, and professional assistance.  • For patients who independently use AI systems, it provides a concise operation guide and learning platform and points out that physicians who use AI require Professional training or the corresponding qualifications.  • It requires time or positive experience to build trust or acceptance of artificial intelligence among patients and physicians.  Suggested adoption: Use alternative assessment methods based on medical benefits, applicability, and ethical considerations  rather than merely focusing on economic factors.  • Advocate for the establishment of transparent quality standards. Clarify the goals and guidelines and discuss the need for regulatory authorities or legal systems to formulate these guidelines.  • It is necessary to establish a supervisory body to monitor artificial intelligence be capable of operation to ensure compliance and clarify the consequences of violations.  • Legislation requires that treatment decisions be made by humans rather than artificial intelligence. And the government's supervision and legal framework in promoting the fair use of artificial intelligence it is necessary to avoid the effect of inhibiting its development |
| Quantitative RCT |  |
| Sebastian et al., 2023 | • Our results indicate that using communication strategies to promote an AI product affects users’ trust, customer innovativeness, and perceived novelty value, leading to improved product adoption. Pathos-laden promotions improve AI product adoption by nudging users’ trust and perceived novelty value of the product Similarly, ethos-laden promotions improve AI product adoption by nudging customer inventiveness. In addition, logos-laden promotions improve AI product adoption by alleviating trust issues |
| Zhou et al., 2022 | not mentioned |
| Quantitative non-randomized |  |
| Chen, 2022 | • Consider the personalized needs of the elderly from different age groups, cultural levels, and family support, and promote affordable, easy-to-operate artificial intelligence robots. |
| Aggarwal et al., 2021 | • Education and Engagement; Enhanced Data Security; Diverse Data Sets; Public Debates and Engagement. |
| Armero et al., 2022 | • Strengthening patient education; Protecting patient privacy and increasing transparency in medical data processing; Enhancing transparency in the decision-making process |
| Ayad et al., 2023 | • Education and Training; Transparency and Trust Building; Addressing Technical Challenges. |
| Baghdadi et al., 2024 | • Efficient implementation of AI requires a good interpretation of the patient’s attitudes toward the use of AI in medicine in order to build their trust. One of the objectives of this study is to understand patient |
| Bahadir et al., 2024 | • Additional education in the area of AI |
| Esin et al., 2024 | not mentioned |
| Esmaeilzadeh et al., 2021 | • Regulatory agencies should establish normative standards and evaluation guidelines for implementing AI in health care in cooperation with health care institutions. Regular audits and ongoing monitoring and reporting systems can be used to continuously evaluate the safety, quality, transparency, and ethical factors of AI clinical applications |
| Fransen et al., 2024 | • Educating patients about the benefits of the current healthcare system • Future collaborative efforts among hospitals, radiologists, AI developers, and legal experts are essential to tailor these adjustments appropriately. |
| Huang et al., 2024 | • Placing further importance on facilitating conditions in the successful implementation of this new pathway |
| Jutzi et al., 2020 | • To encompass the requirement for transparent, AI was also expected to improve processes, and to reduce the burden on healthcare by helping to avoid unnecessary diagnostics and treatments. |
| Kawsar et al., 2023 | • Continued end-user feedback will allow refinement of services to ensure patient acceptability. |
| Khullar et al., 2022 | • Clinicians, policy makers, and developers should be aware of patients’ views regarding AI. Patients may benefit from education on how AI is being incorporated into care and the extent to which clinicians rely on AI to assist with decision-making. Future work should examine how views evolve as patients become more familiar with AI. |
| Kosan et al., 2022 | not mentioned |
| Lennartz et al., 2021 | • Patients strongly preferred physician-controlled application of AI. In order to safeguard patient interests, disclosure and control of AI application in medicine is crucial. |
| Liu et al., 2021 | • The acceptability may be enhanced by choosing and adapting a diagnostic program to cater to patients’ preferences.  • The promotion and spread of AI diagnosis cannot ignore the need to set a suitable diagnosis price or give some discount and bonus according to the wealth status of patients. |
| Mahlknecht et al., 2023 | • The symptom checker could also be further developed to be used by patients in the GPs’ waiting room as a preparation for the medical visit by increasing self-refection. |
| Meyer et al., 2020 | not mentioned |
| Riedl et al., 2024 | • Experimentally manipulate the machine’s characteristics to resemble typical human attributes; |
| Yang, 2019 | • To establish the initial trust by strengthening the education. |
| Parry et al., 2023 | not mentioned |
| Ongena et al., 2020 | • Patients indicated a general need to be well and completely informed on all aspects of the diagnostic process, patients’ need for the development of ethical and legal frame works within which AI systems are allowed to operate. |
| Temple et al., 2023 | • Educate and involve patients in the future direction of this technology；demonstrating to patients that the use of AI in their own healthcare is safe, effective and ethical；establish a baseline educational level. |
| Alsanosi, 2025 | • Patients with chronic diseases are highly aware of the potential of artificial intelligence (AI) to enhance medication compliance through reminders and education, among whom young patients are particularly aware of AI, showing a deeper understanding and a more positive attitude.  • Customized educational strategies for different groups of people-such as special seminars designed for the elderly or digital tools developed for young patients, effectively address demographic differences in KAP.  • Transparent AI series systems (such as those providing data usage instructions or selective consent mechanisms) can alleviate these concerns |
| Bersu Ozcan, 2025 | • By deploying relevant measures, taking mitigation steps, and establishing a patient data protection mechanism.  • Through education, transparency, and a rigorous artificial intelligence verification process, address any concerns. |
| Tirapelli, 2025 | • In conclusion, overall, patients favor the use of AI in dental imaging as an auxiliary tool, with human supervision remaining essential. |
| Chan, 2025 | • The application of AI/ML involves numerous laws and ethics the issues include requirements such as information disclosure, informed consent, and privacy protection. These concerns need to be addressed; the issue can be addressed by the governance of underlying technologies by medical regulatory authorities.  • The active participation of doctors can enhance the patients' trust in the AI/ML systems deployed in healthcare. This kind of participation requires additional training, and education is also strongly demanded by the patients in this study. |
| Quantitative descriptive |  |
| Fu et al., 2023 | • By optimizing the structure of the application and designing user-friendly applications with simple navigation and clear interfaces, usability can be improved, thereby increasing patient acceptance. |
| Liu et al., 2019 | • Strengthen publicity efforts, enhance technology research and development, implement safety certifications, improve the safety and usability of AI in healthcare, establish management systems and standards, and reinforce communication and guidance. |
| Zhang et al., 2022 | • Improve the robot's health education functions, including voice-based Q&A and video consultation features for human-robot interaction.  • Adjust the robot's voice mode to make it as gentle and melodious as possible, with a slow speaking pace. Enhance the patient experience by adding versions with different dialects, allowing patients and their families to switch freely between Mandarin and dialect versions according to their needs. |
| Zhu et al., 2019 | • The government should increase financial investment to support the development of smart healthcare in hospitals; improve relevant laws and regulations, focusing on protecting patient privacy; and ensure top-level design, with hospitals formulating overall plans. |
| Haggenmuller et al., 2024 | not mentioned |
| Macri et al., 2024 | • Highlighted the need for videos to be produced cost-effectively and use able in varying contexts; |
| Mixed methods |  |
| van der Zander et al., 2022 | • Misconceptions and perceived (dis)advantages should be conquered by better disseminating information in layman’s terms and by educating physicians and patients. |
| Katirai et al., 2023 | • There is a growing consensus that consideration is urgently needed of the implications of AI for healthcare prior to its implementation. The meaningful involvement of stakeholders in these processes, including patients and members of the public is essential.  • This study has shown that patients and members of the public are keen to be engaged around AI in healthcare. It is crucial that they be given the opportunity to do so. |
| Gonzalez et al., 2024 | Have official members of the perioperative team deliver patient education  • Communicate the role of ML-CDSS in perioperative care using laymen terms  • Connect patients with in-hospital resources to obtain more information on ML-CDSS (eg, patient navigators)  • Assure patients of the rigor of ML-CDSS training prior to its clinical use • Require perioperative teams to obtain recurrent training on the design and implementation of ML-CDSS  • Require perioperative teams to obtain recurrent ML communications training to better address patients’ technical questions  • Be transparent about costs patients are responsible for related to perioperative use   of ML-CDSS • Be transparent about the specific ML-CDSS’s role in perioperative activities |
| Palmisciano et al., 2020 | not mentioned |
| Robertson et al., 2023 | • when AI was proven to be more accurate；human physicians can support adoption, where the technology is designed with the patient experience in mind and supported by evidence of accuracy. |
| Witkowski et al., 2024 | • providing support with “split-second decisions [that also require] a human touch”  • AI within informed consent procedures and specifically outlining the harms and benefits before using AI with patients. • some of these fears could be addressed by establishing mechanisms for transparency and accountability. |

Note: AI: artificial intelligence; ML-CDSS: Machine Learning-Clinical Decision Support System; GI: gastrointestinal; PwPD: Persons with Parkinson's Disease; SCD: sudden cardiac death; GP: General practitioners; Q&A: Questions and Answer

**Table S10.** Detailed APEASE ratings and feasibility classification of all BCTs.

| UTAUT 2 Component | BCT (v1 Number & Label) | A | P | E | A | S | E | Overall Feasibility | Key Rationale Summary |
| --- | --- | --- | --- | --- | --- | --- | --- | --- | --- |
| Performance Expectancy | 4.1 Instruction on how to perform | 2 | 2 | 2 | 2 | 2 | 1 | High | Low-cost, easily integrated into existing education. |
|  | 6.1 Demonstration of the behavior | 2 | 2 | 2 | 2 | 2 | 1 | High | Use of existing videos/resources is highly practical. |
|  | 5.1 Information about health consequences | 2 | 2 | 2 | 2 | 2 | 1 | High | Core educational component with strong evidence. |
|  | 5.2 Salience of consequences | 1 | 1 | 2 | 1 | 2 | 1 | Medium | Requires targeted design; acceptance may vary. |
|  | 5.3 Anticipated regret | 1 | 1 | 1 | 1 | 2 | 1 | Medium | Limited direct evidence: effect is uncertain. |
| Effort Expectancy | 1.1 Goal setting (behavior) | 2 | 2 | 2 | 2 | 2 | 2 | High | Simple, cost-free, fits well in clinical consultation. |
|  | 2.2 Feedback on behavior | 1 | 1 | 2 | 2 | 2 | 0 | Medium | Effective but depends on technology access (equity concern). |
|  | 2.3 Self-monitoring of behavior | 2 | 2 | 2 | 2 | 2 | 2 | High | Very low-cost, empowers patients, highly practical. |
|  | 2.7 Feedback on outcome(s) of behavior | 1 | 1 | 2 | 2 | 2 | 0 | Medium | Valued but relies on system-generated reports & access. |
|  | 15.1 Verbal persuasion about capability | 1 | 1 | 2 | 1 | 2 | 2 | Medium | Requires training for healthcare providers. |
| Social Influences | 3.1 Social support (unspecified) | 0 | 0 | 2 | 2 | 1 | 2 | Low | Refers to legislation; not implementable by clinical teams. |
|  | 6.3 Information about others' approval | 1 | 1 | 2 | 2 | 2 | 1 | Medium | Requires gathering/disseminating normative data. |
|  | 12.2 Restructuring the social environment (family) | 1 | 1 | 2 | 2 | 2 | 1 | Medium | Involves engaging family, adding coordination. |
| Facilitating Conditions | 2.1 Monitoring of behavior by others without feedback | 1 | 0 | 1 | 0 | 1 | 2 | Low | Ethical/privacy concerns; adds unrewarded workload. |
|  | 9.1 Credible source | 0 | 0 | 2 | 2 | 2 | 2 | Low | Requires institution-level data governance systems. |
|  | 12.1 Restructuring the physical environment | 0 | 0 | 2 | 2 | 2 | 2 | Low | Entails major infrastructure/policy change. |
|  | 12.2 Restructuring the social environment (care relationship) | 1 | 1 | 2 | 2 | 2 | 1 | Medium | Developing institutional guidelines is feasible with support. |
|  | 12.5 Adding objects to the environment | 1 | 0 | 2 | 0 | 2 | 0 | Low | Involves developing new AI features (product development). |
| Hedonic Motivation  Price Value | 5.4 Monitoring of emotional consequences | 1 | 0 | 2 | 1 | 2 | 2 | Medium | Important but requires dedicated provider time. |
|  | 5.6 Information about emotional consequences | 1 | 0 | 2 | 1 | 2 | 2 | Medium | Requires adaptive care based on feedback. |
|  | 1.7 Review outcome goal(s) | 1 | 0 | 2 | 2 | 2 | 1 | Medium | Depends on the AI system's capability for personalized feedback. |
| Habit | 1.1 Goal setting (behavior) | 2 | 2 | 2 | 2 | 2 | 2 | High | (Consistent with BCT 6) |
|  | 1.4 Action planning | 0 | 0 | 2 | 1 | 2 | 2 | Low | Refers to national-level regulatory standards. |
|  | 2.2 Feedback on behavior | 1 | 1 | 2 | 2 | 2 | 0 | Medium | (Consistent with BCT 7) |
|  | 8.3 Habit formation | 1 | 1 | 2 | 2 | 2 | 2 | Medium | Requires establishing structured follow-up protocols. |

Note: This table presents the detailed 3-point APEASE ratings (2 = favorable, 1 = neutral/uncertain, 0 = unfavorable) for each Behavior Change Technique (BCT) identified through the TDF-UTAUT 2 mapping. The Overall Feasibility classification (High, Medium, Low) was derived from the integrated scoring pattern based on the predefined decision rules (see Appendix 5 for rules). A=Affordability, P=Practicality, E=Effectiveness, A=Acceptability, S=Side-effects, E=Equity.

**The list of the included studies**

1. Musbahi, O., et al., *Public patient views of artificial intelligence in healthcare: A nominal group technique study.* Digital Health, 2021. **7**.

2. Armero, W., et al., *A survey of pregnant patients' perspectives on the implementation of artificial intelligence in clinical care.* Journal of the American Medical Informatics Association, 2022. **30**(1): p. 46-53.

3. Godoy Junior, C.A., et al., *Attitudes Toward the Adoption of Remote Patient Monitoring and Artificial Intelligence in Parkinson's Disease Management: Perspectives of Patients and Neurologists.* Patient-Patient Centered Outcomes Research, 2024. **17**(3): p. 275-285.

4. Haan, M., et al., *A Qualitative Study to Understand Patient Perspective on the Use of Artificial Intelligence in Radiology.* J Am Coll Radiol, 2019. **16**(10): p. 1416-1419.

5. Mikkelsen, J.G., et al., *Patient perspectives on data sharing regarding implementing and using artificial intelligence in general practice - a qualitative study.* BMC Health Services Research, 2023. **23**(1).

6. Nelson, C.A., et al., *Patient Perspectives on the Use of Artificial Intelligence for Skin Cancer Screening A Qualitative Study.* Jama Dermatology, 2020. **156**(5): p. 501-512.

7. Ayad, N., et al., *Patients' perspectives on the use of artificial intelligence in dentistry: a regional survey.* Head & Face Medicine, 2023. **19**(1).

8. Esin, H., et al., *Patients' perspectives on the use of artificial intelligence and robots in healthcare.* Bratislava Medical Journal-Bratislavske Lekarske Listy, 2024. **125**(8): p. 513-518.

9. Esmaeilzadeh, P., T. Mirzaei, and S. Dharanikota, *Patients' Perceptions Toward Human-Ar tificial Intelligence Interaction in Health Care: Experimental Study.* Journal of Medical Internet Research, 2021. **23**(11).

10. Jutzi, T.B., et al., *Artificial Intelligence in Skin Cancer Diagnostics: The Patients' Perspective.* Frontiers in Medicine, 2020. **7**.

11. Meyer, A.N.D., et al., *Patient Perspectives on the Usefulness of an Artificial Intelligence-Assisted Symptom Checker: Cross-Sectional Survey Study.* Journal of Medical Internet Research, 2020. **22**(1).

12. Riedl, R., S.A. Hogeterp, and M. Reuter, *Do patients prefer a human doctor, artificial intelligence, or a blend, and is this preference dependent on medical discipline? Empirical evidence and implications for medical practice.* Frontiers in psychology, 2024. **15**: p. 1422177-1422177.

13. Mahlknecht, A., et al., *Supporting primary care through symptom checking artificial intelligence: a study of patient and physician attitudes in Italian general practice.* BMC Prim Care, 2023. **24**(1): p. 174.

14. Yang, K., et al., *Attitudes Of Chinese Cancer Patients Toward The Clinical Use Of Artificial Intelligence.* Patient Preference and Adherence, 2019. **13**: p. 1867-1875.

15. van der Zander, Q.E.W., et al., *Artificial intelligence in (gastrointestinal) healthcare: patients' and physicians' perspectives.* Scientific reports, 2022. **12**(1).

16. Benrimoh, D., et al., *Using a simulation centre to evaluate preliminary acceptability and impact of an artificial intelligence-powered clinical decision support system for depression treatment on the physician-patient interaction.* Bjpsych Open, 2021. **7**(1).

17. Witkowski, K., R. Okhai, and S.R. Neely, *Public perceptions of artificial intelligence in healthcare: ethical concerns and opportunities for patient-centered care.* Bmc Medical Ethics, 2024. **25**(1).

18. Temple, S., C. Rowbottom, and J. Simpson, *Patient views on the implementation of artificial intelligence in radiotherapy.* Radiography (London, England : 1995), 2023. **29 Suppl 1**: p. S112-S116.

19. Zhang, Z., et al., *Patients' perceptions of using artificial intelligence (AI)-based technology to comprehend radiology imaging data.* Health informatics journal, 2021. **27**(2).

20. Sachdeva, M., et al., *Acceptability of artificial intelligence for cervical cancer screening in Dschang, Cameroon: a qualitative study on patient perspectives.* Reproductive Health, 2024. **21**(1).

21. Pelly, M., et al., *Artificial intelligence for secondary prevention of myocardial infarction: A qualitative study of patient and health professional perspectives.* International Journal of Medical Informatics, 2023. **173**.

22. Aggarwal, R., et al., *Patient Perceptions on Data Sharing and Applying Artificial Intelligence to Health Care Data: Cross-sectional Survey.* Journal of Medical Internet Research, 2021. **23**(8).

23. Sebastian, G., A. George, and G. Jackson, *Persuading Patients Using Rhetoric to Improve Artificial Intelligence Adoption: Experimental Study.* J Med Internet Res, 2023. **25**: p. e41430.

24. Lingli, L., H. Yimo, and L. Xiangde, *Investigation on Patients’ Cognition and Trust in Artificial Intelligence Medicine.* Chinese Medical Ethics, 2019. **32**(8).

25. Change, Z., Z. Huiling, and Z. Huiyin, *Study on the Influence Mechanism of the Use of Hospital Intelligent Medical System on Patients’Satisfaction：Based on the Perspective of Technology Acceptance Model.* Chinese Hospital Management, 2019. **39**(10): p. 61-64.

26. Zhou, Y., et al., *Did Artificial Intelligence Invade Humans? The Study on the Mechanism of Patients' Willingness to Accept Artificial Intelligence Medical Care: From the Perspective of Intergroup Threat Theory.* Frontiers in psychology, 2022. **13**: p. 866124-866124.

27. Adams, S.J., R. Tang, and P. Babyn, *Patient Perspectives and Priorities Regarding Artificial Intelligence in Radiology: Opportunities for Patient-Centered Radiology.* Journal of the American College of Radiology, 2020. **17**(8): p. 1034-1036.

28. Robinson, R., et al., *Artificial Intelligence in Health Care-Understanding Patient Information Needs and Designing Comprehensible Transparency: Qualitative Study.* Jmir Ai, 2023. **2**: p. e46487-e46487.

29. Baghdadi, L.R., et al., *Patients' Attitudes Toward the Use of Artificial Intelligence as a Diagnostic Tool in Radiology in Saudi Arabia: Cross-Sectional Study.* JMIR human factors, 2024. **11**: p. e53108-e53108.

30. Khullar, D., et al., *Perspectives of Patients About Artificial Intelligence in Health Care.* JAMA Network Open, 2022. **5**(5).

31. Parry, M.W.W., et al., *Patient Perspectives on Artificial Intelligence in Healthcare Decision Making: A Multi-Center Comparative Study.* Indian Journal of Orthopaedics, 2023. **57**(5): p. 653-665.

32. Pelayo, C., et al., *Perspectives of Latinx Patients with Diabetes on Teleophthalmology, Artificial Intelligence-Based Image Interpretation, and Virtual Care: A Qualitative Study.* Telemedicine reports, 2023. **4**(1): p. 317-326.

33. Bahadir, H.S., et al., *Patients' attitudes toward artificial intelligence in dentistry and their trust in dentists.* Oral Radiology, 2024.

34. Lennartz, S., et al., *Use and Control of Artificial Intelligence in Patients Across the Medical Workflow: Single-Center Questionnaire Study of Patient Perspectives.* Journal of Medical Internet Research, 2021. **23**(2).

35. ZHANG Siwen, L.D., DUAN Gaoyang, ZHONG Jiudi, *Investigation on the Acceptance of Health Education Robots among Patients, Family Members, and Medical Staff in Esophageal Cancer Surgery.* Modern Nurse · Special Edition, 2022. **29**(1).

36. Siwen, Z., et al., *A survey on the acceptance of health education robots by esophageal cancer surgery patients, their families and healthcare workers.* Modern Nurse, 2022. **29**(1).

37. Gonzalez, X.T., K. Steger-May, and J. Abraham, *Just another tool in their repertoire: uncovering insights into public and patient perspectives on clinicians' use of machine learning in perioperative care.* Journal of the American Medical Informatics Association, 2024.

38. Haggenmuller, S., et al., *Patients' and dermatologists' preferences in artificial intelligenceedriven skin cancer diagnostics: A prospective multicentric survey study.* Journal of the American Academy of Dermatology, 2024. **91**(2): p. 366-370.

39. Ongena, Y.P., et al., *Patients' views on the implementation of artificial intelligence in radiology: development and validation of a standardized questionnaire.* European Radiology, 2020. **30**(2): p. 1033-1040.

40. Kai, W., et al., *Robot-assisted Gait Training Experience in Stroke Patients: A Qualitative Research.* Military Nursing, 2023. **40**(2).

41. Katirai, A., et al., *Perspectives on artificial intelligence in healthcare from a Patient and Public Involvement Panel in Japan: an exploratory study.* Frontiers in Digital Health, 2023. **5**: p. 1229308-1229308.

42. Jalil, S., et al., *Complementing a Clinical Trial With Human-Computer Interaction: Patients' User Experience With Telehealth.* JMIR human factors, 2019. **6**(2): p. e9481-e9481.

43. Maris, M.T., et al., *Ethical use of artificial intelligence to prevent sudden cardiac death: an interview study of patient perspectives.* Bmc Medical Ethics, 2024. **25**(1).

44. WANG Kai, J.A., LIYan, YANG Xiaopei, FEI Wenling, YU Xiaoli, MENG Guilin, ZHU Xiaoping, *Robot-assisted Gait Training Experience in Stroke Patients: A Qualitative Research*, in *military nursing*. 2023.

45. Fransen, S.J., et al., *Patient perspectives on the use of artificial intelligence in prostate cancer diagnosis on MRI.* European Radiology, 2024.

46. Liu, T., et al., *Patients' Preferences for Artificial Intelligence Applications Versus Clinicians in Disease Diagnosis During the SARS-CoV-2 Pandemic in China: Discrete Choice Experiment.* Journal of Medical Internet Research, 2021. **23**(2).

47. Xiangde, L.L.H.Y.L., *Investigation on Patients’Cognition and Trust in Artificial Intelligence Medicine.* Chinese Medical Ethics, 2019. **32**(8).

48. Kawsar, A., et al., *Patient perspectives of artificial intelligence as a medical device in a skin cancer pathway.* Frontiers in Medicine, 2023. **10**.

49. Huang, W., et al., *Applying the UTAUT2 framework to patients' attitudes toward healthcare task shifting with artificial intelligence.* BMC Health Services Research, 2024. **24**(1).

50. Shuojin, F., et al., *Development and acceptance of a virtual reality system for rehabilitation training of swallowing disorders in stroke patients.* Chinese Journal of Modern Nursing, 2023. **29**(18): p. 2421-2426.

51. Macri, C.Z., et al., *A pilot survey of patient perspectives on an artificial intelligence-generated presenter in a patient information video about face-down positioning after vitreoretinal surgery.* Ophthalmic research, 2024: p. 1-12.

52. Palmisciano, P., et al., *Attitudes of Patients and Their Relatives Toward Artificial Intelligence in Neurosurgery.* World Neurosurg, 2020. **138**: p. e627-e633.

53. Kosan, E., et al., *Patients' Perspectives on Artificial Intelligence in Dentistry: A Controlled Study.* Journal of Clinical Medicine, 2022. **11**(8).

54. Robertson, C., et al., *Diverse patients' attitudes towards Artificial Intelligence (AI) in diagnosis.* PLOS digital health, 2023. **2**(5): p. e0000237-e0000237.

55. Yulan, Z., et al., *Willingness of elderly patients to use artificial intelligence robots and its influencing factors.* West China Medical Journal, 2022. **37**(9).
