# Supplementary material for: Barriers and Facilitators to Patient Acceptance of Artificial Intelligence in Health Care: Systematic Review
Source: J Med Internet Res. 2026 May 8;28:e80581. doi: 10.2196/80581 (PMC13154726; doi:10.2196/80581)
Supplement: Checklist 1 [file jmir-v28-e80581-s002.docx]

| **Section/topic** | **#** | **Checklist item** | **Location(s) Reported** |
| --- | --- | --- | --- |
| **INFORMATION SOURCES AND METHODS** | | | |
| Database name | 1 | Name each individual database searched, stating the platform for each. | **Page7、Appendix 1** |
| Multi-database searching | 2 | If databases were searched simultaneously on a single platform, state the name of the platform, listing all of the databases searched. | **Appendix 1** |
| Study registries | 3 | List any study registries searched. | **Appendix 1** |
| Online resources and browsing | 4 | Describe any online or print source purposefully searched or browsed (e.g., tables of contents, print conference proceedings, web sites), and how this was done. | **Page7、Appendix 1** |
| Citation searching | 5 | Indicate whether cited references or citing references were examined, and describe any methods used for locating cited/citing references (e.g., browsing reference lists, using a citation index, setting up email alerts for references citing included studies). | **Page 7** |
| Contacts | 6 | Indicate whether additional studies or data were sought by contacting authors, experts, manufacturers, or others. | **Page 8** |
| Other methods | 7 | Describe any additional information sources or search methods used. | **Page 7** |
| **SEARCH STRATEGIES** | | | |
| Full search strategies | 8 | Include the search strategies for each database and information source, copied and pasted exactly as run. | **Appendix 1** |
| Limits and restrictions | 9 | Specify that no limits were used, or describe any limits or restrictions applied to a search (e.g., date or time period, language, study design) and provide justification for their use. | **Appendix 1** |
| Search filters | 10 | Indicate whether published search filters were used (as originally designed or modified), and if so, cite the filter(s) used. | **Appendix 1** |
| Prior work | 11 | Indicate when search strategies from other literature reviews were adapted or reused for a substantive part or all of the search, citing the previous review(s). | **None** |
| Updates | 12 | Report the methods used to update the search(es) (e.g., rerunning searches, email alerts). | **Page 7** |
| Dates of searches | 13 | For each search strategy, provide the date when the last search occurred. |  |
| **PEER REVIEW** | | | |
| Peer review | 14 | Describe any search peer review process. | **Page 7** |
| **MANAGING RECORDS** | | | |
| Total Records | 15 | Document the total number of records identified from each database and other information sources. | **Figure_1、Page 9** |
| Deduplication | 16 | Describe the processes and any software used to deduplicate records from multiple database searches and other information sources. | **Figure_1 、****Page 9** |
|  |  |  |  |
| PRISMA-S: An Extension to the PRISMA Statement for Reporting Literature Searches in Systematic Reviews | | |  |
| Rethlefsen ML, Kirtley S, Waffenschmidt S, Ayala AP, Moher D, Page MJ, Koffel JB, PRISMA-S Group. | | |  |
| Last updated February 27, 2020. | |  |  |
